# Viral DNA Replication Orientation and hnRNPs Regulate Transcription of the Human Papillomavirus 18 Late Promoter

**DOI:** 10.1128/mBio.00713-17

**Published:** 2017-05-30

**Authors:** Xiaohong Wang, Haibin Liu, Hui Ge, Masahiko Ajiro, Nishi R. Sharma, Craig Meyers, Pavel Morozov, Thomas Tuschl, Amar Klar, Donald Court, Zhi-Ming Zheng

**Affiliations:** a RNA Biology Laboratory, Center for Cancer Research, National Cancer Institute, National Institutes of Health, Frederick, Maryland, USA; b AscentGene, Inc., Gaithersburg, Maryland, USA; c Department of Microbiology and Immunology, Penn State University College of Medicine, Hershey, Pennsylvania, USA; d Howard Hughes Medical Institute and Laboratory of RNA Molecular Biology, Rockefeller University, New York, New York, USA; Icahn School of Medicine at Mount Sinai

**Keywords:** DNA replication, HPV18, human papillomaviruses, gene expression, promoters, small RNAs, transcription, transcriptional regulation

## Abstract

The life cycle of human papillomaviruses (HPVs) is tightly linked to keratinocyte differentiation. Although expression of viral early genes is initiated immediately upon virus infection of undifferentiated basal cells, viral DNA amplification and late gene expression occur only in the mid to upper strata of the keratinocytes undergoing terminal differentiation. In this report, we show that the relative activity of HPV18 TATA-less late promoter P_811_ depends on its orientation relative to that of the origin (Ori) of viral DNA replication and is sensitive to the eukaryotic DNA polymerase inhibitor aphidicolin. Additionally, transfected 70-nucleotide (nt)-long single-strand DNA oligonucleotides that are homologous to the region near Ori induce late promoter activity. We also found that promoter activation in raft cultures leads to production of the late promoter-associated, sense-strand transcription initiation RNAs (tiRNAs) and splice-site small RNAs (spliRNAs). Finally, a *cis*-acting AAGTATGCA core element that functions as a repressor to the promoter was identified. This element interacts with hnRNP D0B and hnRNP A/B factors. Point mutations in the core prevented binding of hnRNPs and increased the promoter activity. Confirming this result, knocking down the expression of both hnRNPs in keratinocytes led to increased promoter activity. Taking the data together, our study revealed the mechanism of how the HPV18 late promoter is regulated by DNA replication and host factors.

## IMPORTANCE

It has been known for decades that the activity of viral late promoters is associated with viral DNA replication among almost all DNA viruses. However, the mechanism of how DNA replication activates the viral late promoter and what components of the replication machinery are involved remain largely unknown. In this study, we characterized the P_811_ promoter region of HPV18 and demonstrated that its activation depends on the orientation of DNA replication. Using single-stranded oligonucleotides targeting the replication fork on either leading or lagging strands, we showed that viral lagging-strand replication activates the promoter. We also identified a transcriptional repressor element located upstream of the promoter transcription start site which interacts with cellular proteins hnRNP D0B and hnRNP A/B and modulates the late promoter activity. This is the first report on how DNA replication activates a viral late promoter.

## INTRODUCTION

Persistent infection by high-risk (HR) human papillomavirus (HPV) types leads to development of cervical cancer, the second leading cause of death among women worldwide (1, 2). The HR HPVs also cause genital cancers, such as cancers of the anus, vulva, vagina, and penis, and cause a fraction of head and neck cancers (3). HPVs are small DNA viruses. Their genomes are circular and approximately 8 kb in size and encode eight major proteins, six (E1, E2, E4, E5, E6, and E7) located in the “early” region and two (L1 and L2 capsid proteins) in the “late” region (4). The early proteins regulate viral and host gene expression and play roles in viral DNA maintenance and amplification and pathogenesis. L1 and L2 form the virus capsid required for virus morphogenesis, transmission, and survival under the various environmental conditions (4).

HPVs infect the basal stratum of stratified squamous epithelia, and their life cycle is tightly linked to the differentiation state of the keratinocytes. HPV expresses viral early genes in the undifferentiated basal/parabasal layers and mid-spinous strata and late genes in the upper spinous cells undergoing terminal differentiation. Characterization of various papillomaviruses indicates that the late mRNAs of bovine papillomavirus 1 (BPV-1) (5), HPV6 (6), HPV11 (6), HPV16 (7), and HPV31 (8) are activated in differentiated keratinocytes. Although cell differentiation signals alone may activate transcription from the HPV31 late promoter (LP) P_742_ to some extent, viral DNA replication is required for maximal activity of the late promoter (9, 10) and for late protein expression (11, 12). A link between late gene expression and vegetative DNA replication has also been well established for adenovirus (13–15), simian virus 40 (SV40) (16), polyomaviruses (17, 18), herpes simplex virus (HSV) (19), cytomegalovirus (CMV) (20), Kaposi's sarcoma-associated herpesvirus (KSHV) (21), and poxvirus (22) and even for bacteriophages (23–25). However, how the late promoter is induced by viral DNA replication in many viruses remains largely unknown.

The HPV genome contains a single origin of DNA replication, called Ori. Replication of the HPV genome is carried out by host replication machinery along with two viral replication proteins, E1 and E2 (26). The E1 protein is an ATP-dependent DNA helicase and assembles on the Ori as a dimer of hexamers, forming a bidirectional helicase (27, 28). The helicase is required for viral DNA replication initiation and is also necessary throughout the replication elongation process (29, 30). E2 is the Ori recognition protein and interacts with E1 to enhance E1 binding affinity and specificity with respect to the Ori (31); it is not required during viral DNA replication. HPV DNA replication is initiated bidirectionally. Both HPV16 and HPV31 DNA replication products in undifferentiated cells primarily consist of theta structures. Theta replication converts to asymmetric, unidirectional rolling-circle replication when the cells are grown under culture conditions that promote epithelial cell differentiation (32). Human epithelial cell extract supplemented with purified E1 and E2 supports rolling-circle replication of HPV16 Ori-containing plasmid DNA (33). Rolling-circle replication was proposed as the mechanism for BPV-1 DNA replication (34, 35), and replicative intermediates containing single forks with a mass greater than that of half of the replicated molecules were observed in HPV11 DNA replication (36). A recent study using an osteosarcoma cell line demonstrated that HPV genomes exert an alternative, recombination-dependent replication mechanism (37, 38); however, whether this mode of replication occurs in differentiating keratinocytes is not known. The late mRNAs of various HPVs have been shown to be derived from a promoter within the E7 open reading frame (ORF) (39–42). We recently mapped the HPV18 late promoter transcription start site (TSS) at nucleotide (nt) position 811 in the E7 ORF during productive HPV18 infection (43). In this study, we characterized the P_811_ promoter region of HPV18 and demonstrated that promoter activity depends largely on the direction of DNA replication. We also identified a transcriptional repressor element located upstream of the promoter that interacts with cellular proteins hnRNP D0B and hnRNP A/B to affect transcriptional repression.

## RESULTS

### Characterization of HPV18 late promoter P_811_ activity and its relation to the viral Ori and cell differentiation.

**FIG 1:**
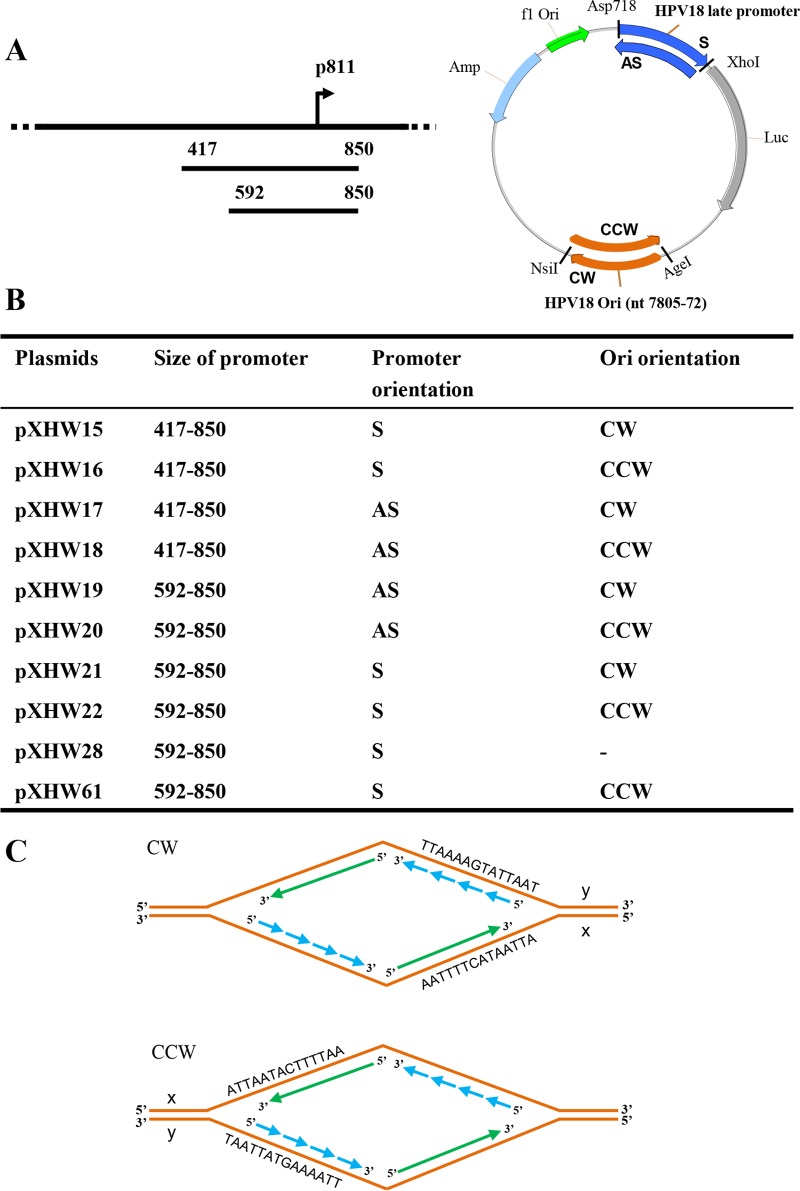
Construction of HPV18 late promoter- and replication origin-containing plasmids. (A) A line diagram of the HPV18 genome with the relative position of the P_811_ late promoter TSS (arrow) indicated. Below the genome are two versions (a longer version from nt 417 to nt 850 and a shorter version from nt 592 to nt 850) of the putative late-promoter region running over the TSS inserted at Asp718 and XhoI sites upstream of a *firefly* luciferase gene in either a sense (S) or an antisense (AS) orientation (see the map on the right). The minimal HPV18 replication origin (HPV18 Ori) from nt 7805 to nt 72 (124 bp) (44, 45) in the circular HPV18 genome was inserted in either a clockwise (CW) or a counterclockwise (CCW) orientation (relative to the plasmid map) into AgeI and NsiI sites downstream of the Luc ORF (see the map). The plasmid illustration is not drawn to scale. (B) Plasmid names and features are listed. (C) Diagrams of the replication forks derived from each replication Ori in the indicated orientation. Solid arrows, leading strand; dashed arrows, lagging strand; ATTAATACTTTTAA, a representative sequence from the HPV18 Ori; x and y, two DNA strands of the Ori in CW or CCW orientation relative to the reporter.

**FIG 2:**
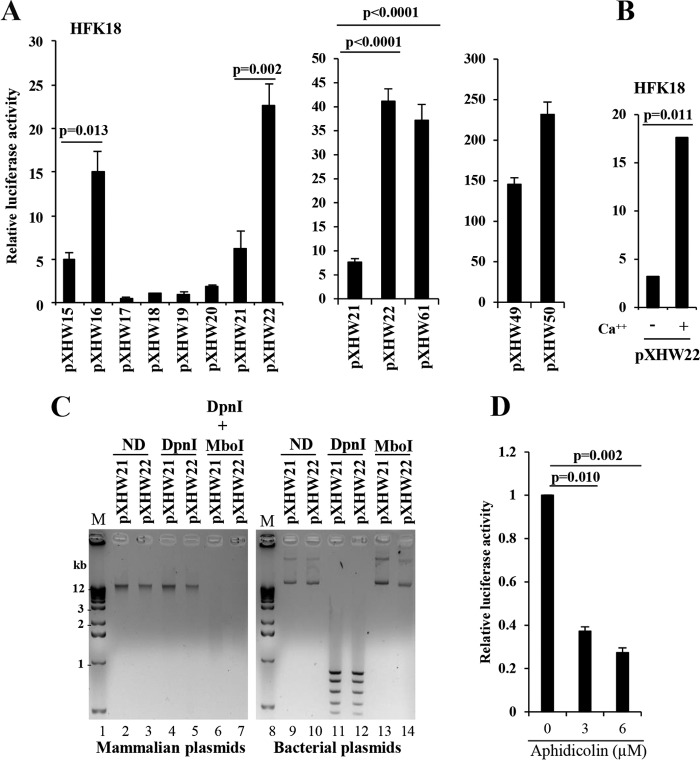
Characterization of the late-promoter region of HPV18. (A) The strong activity of a P_811_ promoter region is dependent on CCW orientation of the HPV18 Ori. HPV18-infected human foreskin keratinocytes (HFK18), differentiated by adding 2 mM calcium, were transfected for 48 h with each of the indicated plasmids. (A) *Renilla* luciferase plasmid pRL-TS was cotransfected as an internal control. The supernatant of the cell lysates from each transfection was examined for dual luciferase activities. Relative promoter activity levels were calculated by dividing the value representing the light unit readings from a testing promoter-*firefly* luciferase reporter by the value representing the light unit readings from the *Renilla* luciferase reporter (left panel). Plasmid pXHW61 directly derived from pXHW21 has the HPV18 Ori flipped into a CCW orientation (middle panel). Plasmid pXHW21-derived pXHW49 and pXHW22-derived pXHW50 have their corresponding promoter regions replaced by the SV40 early promoter derived from the pGL3 control vector (right panel). The data shown are means ± standard deviations (SD) of results from two to three independent experiments. *P* values were calculated using Student’s *t* test. (B) The P_811_ promoter activity depends on keratinocyte differentiation. HFK18 cells with (+) or without (−) 2.0 mM calcium treatment were transfected with pXHW22 for 48 h and then analyzed for their luciferase activity. The data shown are from results from one of two experiments, with means ± SD calculated from triplicate samples. (C) Opposite orientations of the HPV18 Ori do not affect plasmid DNA replication in HFK18 cells. HPV18-infected HFK cells, differentiated by 2 mM calcium, were transfected with pXHW21 (Ori in CW orientation) or pXHW22 (Ori in CCW orientation) for 48 h. Replicated plasmid DNA isolated from the cells and the original input bacterial plasmid DNA were compared for their sensitivity to DpnI (digesting only methylated bacterial plasmid DNA) and MboI (digesting only unmethylated bacterial and replicated plasmid DNA). Because the input bacterial DNA is methylated at the adenine of GATC sequences, it is sensitive to digestion by DpnI but resistant to digestion by MboI (right panel), and because human cells lack adenine methylase activity, the newly replicated DNA lacking adenine methylation is thus resistant to DpnI digestion but susceptible to MboI digestion (left panel). ND, no digestion with a restriction enzyme. The digested DNA samples were then resolved in a 1% agarose gel and imaged by ethidium bromide staining. Lanes 1 and 8 represent DNA markers (M). (D) Aphidicolin, a DNA polymerase inhibitor, blocks CCW orientation-dependent HPV18 late promoter activity. The sensitivity of Ori-directed DNA replication and HPV18 late promoter activity in plasmid pXHW22 to aphidicolin at different doses was analyzed in the HFK18 cells cotransfected with plasmids pXHW22 and pRL-SV40 (an internal control). The supernatant of the cell lysates was examined for dual luciferase activities, and the relative promoter activity levels were calculated as described for panel A.

In a previous study, we demonstrated that HPV18 late transcription has heterogeneous transcription start sites but exhibits a dominant start site at nt 811 in the genome. To characterize further the P_811_ promoter activity, we constructed a series of plasmids containing a luciferase (luc) reporter (Fig. 1A and B; see also Fig. S1 in the supplemental material). We inserted either a long (nt 417 to 850) or a short (nt 592 to 850) fragment of HPV18 sequence containing the region at position 811 upstream of a luc gene in either a sense (S) or an antisense (AS) orientation relative to the luc gene. We also inserted the HPV18 Ori (nt 7805 to 7857 and nt 1 to 72) (44, 45) into the reporter plasmid downstream of the luc ORF in either a clockwise (CW) or a counterclockwise (CCW) orientation (Fig. 1B and C; Fig. S1) relative to the plasmid backbone to examine how the viral late promoter activity requires viral DNA replication (9, 14, 18, 20, 21, 25). It is known that the HPV18 Ori also overlaps the early P_55_ promoter such that the TATA box for the early P_55_ promoter is within the A+T-rich region of the core Ori (Fig. S2A) (43, 46). Insertion of the Ori bearing the early P_55_ promoter upstream of the late P_811_ promoter would overwhelm the TATA-less late P_811_ promoter activity. Thus, we inserted the Ori-P_55_ DNA downstream of the luc gene to prevent an Ori effect on the late promoter P_811_ (Fig. S2A). Our strategy was to dissociate the viral early P_55_ promoter activity from the viral late P_811_ promoter activity by using HPV18-immortalized primary human foreskin keratinocytes (HFK18 cells), which allow viral E1 and E2 expression (Fig. S3), being necessary for the viral Ori activity (30, 47, 48). As shown in Fig. 2A, pXHW16 and pXHW22 plasmids, which contain the HPV18 Ori in CCW orientation on either the long or short version of the late promoter cloned in sense orientation, displayed promoter activities from a transcription start site predominantly at P_811_ in HFK18 cells that were much higher than those seen with the equivalent plasmids (pXHW15 and pXHW21) with the Ori in the CW orientation relative to the late promoter as found in the HPV18 genome. The findings were surprising because this orientation of the Ori relative to the late promoter in pXHW16 and pXHW22 is opposite what is normally found in the natural HPV18 genome. The late promoter in the antisense orientation relative to the luc gene showed little if any activity (pXHW17, pXHW18, pXHW19, and pXHW20), regardless of the Ori orientation. As expected, insertion of an Ori carrying the early P_55_ promoter TATA box in either the CW or CCW orientation immediately upstream of the P_811_ promoter occluded the Ori dependence of the late promoter activity (Fig. S1 and S2B). This observation could be further confirmed by insertion of a synthetic poly(A) signal upstream of the reporter’s late P_811_ promoter but downstream of the Ori-containing region, including E6 ORF (nt 7805 to 591) (Fig. S1 and S2B).

10.1128/mBio.00713-17.1 FIG S1  Representative plasmids constructed to detect HPV18 late promoter activity in luciferase reporter assays. (A) A line diagram of the HPV18 genome with the relative position of the late promoter P_811_ (arrow). Below the genome are two versions (a longer version [nt 417 to nt 850] and a shorter version [nt 592 to nt 850]) of the putative late-promoter region running over the TSS P_811_ site being inserted at Asp718 and XhoI sites upstream of a *firefly* luciferase gene. (B) Each plasmid contains a short version (nt 592 to nt 850) of the HPV18 late promoter inserted in either antisense (pXHW19 and pXHW20) or sense (pXHW21 and pXHW22) orientation, along with an HPV18 Ori inserted at AgeI and NsiI sites in either clockwise (CW; pXHW19 and pXHW21) or counterclockwise (CCW; pXHW20 and pXHW22) orientation. (C) Plasmid maps of pXHW21-derived pXHW49 and pXHW22-derived pXHW50 with their corresponding HPV18 promoter regions being replaced by an SV40 early promoter derived from a pGL3 control vector. (D) Plasmid maps of pMA102, pMA103, pHBL10, and pHBL11. Plasmid pMA102 has the Ori in CW orientation and pMA103 has the Ori in CCW orientation immediately upstream of the late promoter P_811_, whereas pHBL10 and pHBL11 have insertions of the HPV18 E6 ORF plus a synthetic poly(A) signal immediately downstream of the Ori as an addition to separate the Ori from the P_811_ late promoter. Download FIG S1, PDF file, 0.1 MB. Copyright © 2017 Wang et al. 2017 Wang et al. This content is distributed under the terms of the Creative Commons Attribution 4.0 International license.

10.1128/mBio.00713-17.2 FIG S2  HPV18 core Ori and its relation to viral early promoter P_55_ and late promoter P_811_. (A) HPV18 genome sequence from its core Ori to the E6 ORF region, with the sequence underlined for HPV18 core Ori, orange letters for three E2 binding sites (E2BS), red letters for two TATA boxes, green letters for transcription start sites (TSS) at nt 55 and nt 102 in the virus genome, and italic letters for E6 ORF with translation initiation codon ATG bolded. (B) HPV18 early promoter P_55_ in the core Ori immediately upstream of the late promoter in pMA102 and pMA103 overwhelms the late promoter P_811_ activity in the Luc reporter assays, but this effect on the P_811_ late promoter can be blocked by insertion of a synthetic poly(A) signal immediately downstream. See details in Fig. 2A for cotransfection of HFK18 cells and measurement of late promoter activity at 24 h posttransfection. (C) Activity of HPV18 late promoter in HPV16-positive W12 subclone 20863 cells and HPV-negative HEK293 cells. Plasmids pXHW16, pXHW18, pXHW22, and pXHW28 were transfected into W12 subclone 20863 cells (an HPV16^+^ cervical cell line generated from low-grade squamous intraepithelial lesion which contains an episomal form of HPV16 genome) or HEK293 cells, and their promoter activities were examined at 48 h after transfection as described for Fig. 2A. Download FIG S2, PDF file, 0.1 MB. Copyright © 2017 Wang et al. 2017 Wang et al. This content is distributed under the terms of the Creative Commons Attribution 4.0 International license.

10.1128/mBio.00713-17.3 FIG S3  Detection of HPV18 E1 and E2 in calcium-induced HFK18 cells by RT-PCR. Total RNA from HFK18 cells was examined by RT-PCR using an E1 primer set consisting of oZMZ252 and oZMZ229 or an E2 primer set consisting of oXHW46 and oMA97 (Table S2). RT, reverse transcriptase; M, a 100-bp DNA ladder; calcium low and high data indicate the HFK18 cells grown in calcium-free (low) or calcium (2 mM)-containing (high) EpiLife medium. Download FIG S3, PDF file, 0.03 MB. Copyright © 2017 Wang et al. 2017 Wang et al. This content is distributed under the terms of the Creative Commons Attribution 4.0 International license.

Because the pXHW22 containing the Ori in CCW orientation displayed higher promoter activity than pXHW21 containing the Ori in CW orientation, we excised and reinserted the Ori from pXHW21 to generate the CCW orientation to confirm the Ori orientation effect. We found that the new plasmid, pXHW21-derived pXHW61, exhibited high promoter activity, comparable to that of pXHW22 (Fig. 2A, middle panel). Thus, the weak activity of the late promoter in pXHW21 was not due to unknown DNA sequence errors introduced into the plasmid; rather, it was due to the CW orientation of the Ori. These results indicate that efficient promoter activity depends on DNA replication by the Ori existing in the CCW orientation. This effect seems to be highly late promoter specific (~4.5-fold increase) as the activity of an SV40 early promoter had only a weak response (~0.5-fold increase) to the HPV Ori orientation. When the HPV18 late promoter in the corresponding pXHW21 and pXHW22 plasmids was replaced by an SV40 early promoter, pXHW21-derived pXHW49 and pXHW22-derived pXHW50 plasmids exhibited little (~0.5-fold) difference in the levels of their responses to the HPV18 Ori orientation (Fig. 2A, right panel). More importantly, when the levels of late promoter activity of pXHW22 in HFK18 cells were compared in the presence or absence of 2 mM calcium, we found that its activity was significantly higher in the presence of calcium (Fig. 2B), indicating that its activity depends on cell differentiation and viral DNA replication induced by calcium treatment. Furthermore, we found that the Ori orientation-dependent HPV18 late promoter activity in pXHW16 and pXHW22 was also observed in W12 subclone 20863 cells harboring an episomal HPV16 genome (49) (Fig. S2C). In HEK293 cells, HPV18 late promoter activity appeared to be independent of the Ori orientation and comparable to that seen with a reporter lacking the Ori (Fig. S2C; compare pXHW22 to pXHW28) or to that seen with a promoter in the antisense orientation (Fig. S2C; compare pXHW16 to pXHW18). Together, these data provide further evidence that expression of viral proteins E1 and E2 from an extrachromosomal HPV genome is necessary for viral DNA replication and for the late promoter’s regulation. Consistent with our observation, earlier reports had indicated that viral E1 and E2 proteins support replication of homologous and heterologous papillomavirus Ori-containing plasmids (50, 51).

### The two HPV18 Ori orientations function equally well for replication of plasmids in HFK18 keratinocytes.

Next, we examined whether the HPV18 Ori orientation affects the efficiency of the plasmid pXHW21 and pXHW22 replication that contributes to different promoter activities in HFK18 cells. To do this, pXHW21 and pXHW22 were transfected into HFK18 cells for 48 h in the presence of 2 mM calcium. The replicated plasmid DNA isolated from the transfected HFK18 cells was compared with its corresponding bacterial plasmid input for sensitivity to digestion by DpnI and MboI endonucleases. Plasmids replicating in mammalian cells lose the adenine base methylation of the transfected DNA, and become resistant to DpnI digestion but susceptible to MboI digestion. This procedure distinguished replicated plasmid DNA in HFK18 cells from the input bacterial plasmid DNA. The latter is methylated at adenine in the GATC restriction site that is susceptible to DpnI but resistant to MboI. As shown in Fig. 2C, pXHW21 and pXHW22 isolated from HFK18 cells were almost equally resistant to DpnI digestion but were equally digestible by MboI. In contrast, pXHW21 and pXHW22 isolated from bacteria and used for transfection of HFK18 cells were efficiently digested by Dpn1 but resistant to MboI digestion. These data indicate that pXHW21 and pXHW22 could replicate equally well in HFK18 cells irrespective of the Ori orientation. Thus, the effect of Ori on the observed transcription cannot be due to an indirect effect of Ori on plasmid replication. More supportively, in separate experiments, we further demonstrated that the Ori-dependent promoter activity in the pXHW22 plasmid could be blocked by aphidicolin (52), a DNA polymerase inhibitor (Fig. 2D).

### DNA strand specificity affects HPV18 Ori-activated promoter activity.

DNA replication occurs on both DNA strands in opposite directions at the replication fork. The leading strand is replicated by DNA polymerase continuously in the direction of replication, whereas the lagging strand is replicated discontinuously in rather short segments by another polymerase (Fig. 1C). In bacteria, single-strand oligonucleotides have been shown to target the replication fork on both leading and lagging strands to generate genomic recombinants. Targeting of the discontinuous lagging strand by a specific oligonucleotide is 20-fold more effective than targeting of the leading strand by a complementary oligonucleotide. This is perhaps indicative of the greater single-strandedness of the lagging-strand DNA template. In *Escherichia coli* bacteria, the rate of lagging-strand recombination, the process called recombineering, is very high, with frequencies approaching 50% of all cells treated with the lagging-strand oligonucleotide (53–55).

**FIG 3:**
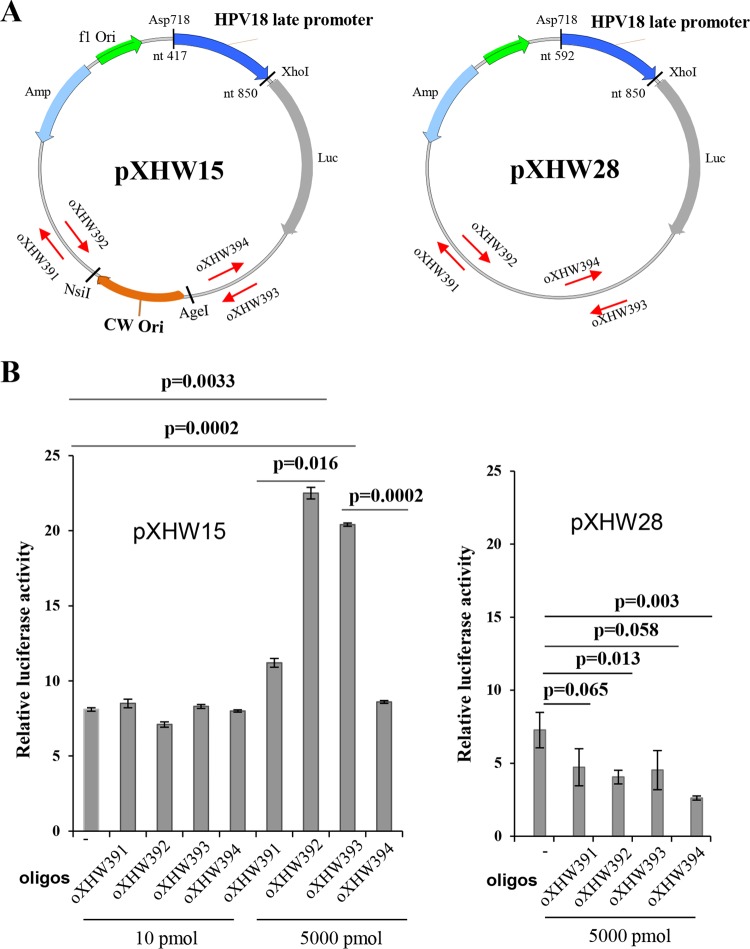
Strand-biased effect on HPV18 Ori-directed DNA replication and HPV18 late promoter activity revealed by a single-stranded, 70-nt-long DNA oligonucleotide. (A) The maps of plasmids pXHW15 (left panel) and pXHW28 (right panel) and the relative positions and orientations (arrow directions) of paired oXHW391/oXHW392 and oXHW393/oXHW394 oligonucleotides. (B) Effects of individual oligonucleotides (oligos) on promoter activity in pXHW15- and pXHW28-transfected HFK18 cells. HFK18 cells were cotransfected with pXHW15 or pXHW28 at the indicated doses of individual oligonucleotides, along with plasmid pRL-TS, and were cultured in a complete culture medium supplemented with 2.0 mM calcium. Dual luciferase activities were analyzed and calculated at 48 h after transfection.

We employed such an oligonucleotide approach to examine whether the asymmetry of HPV18 DNA replication-mediated late promoter activation might be explained by the Ori orientation with respect to leading-strand versus lagging-strand replication at the promoter region. We designed two pairs of complementary oligonucleotides, each 70 nt long and homologous to vector DNA, with one set targeting each side of the HPV18 Ori (Fig. 3A). Each oligonucleotide was cotransfected by electroporation into HFK18 cells along with plasmid pXHW15, which contains the HPV18 Ori in the CW orientation. Plasmid pXHW28, which contains no viral Ori, served as a control. The transfected cells were incubated for 48 h, and the levels of promoter activity were compared for the two plasmids by luciferase assay. We found that both antisense oligonucleotide oXHW392 and sense oligonucleotide oXHW393 (relative to the late promoter) on either side of the pXHW15 Ori in CW orientation could promote viral late promoter activity at 5,000 pM but not at 10 pM (Fig. 3B). This was a surprising result, because pXHW15 displayed little promoter activity without oligonucleotide treatment (Fig. 3B). The two oligonucleotides that activated pXHW15 transcription corresponded to the lagging-strand oligonucleotides, while the leading-strand oligonucleotide treatment had no effect. This DNA strand-bias effect on the late promoter activity suggested that the oXHW392 and oXHW393 oligonucleotides might enter the nucleus and base pair with the replicating plasmid DNA to affect lagging-strand elongation, resulting in asymmetric DNA elongation in one or the other direction to drive the late promoter activity in this reporter plasmid. The same set of oligonucleotides had no effect at 5,000 pM on promoter activity in pXHW28 lacking a viral origin (Fig. 3B).

### Identification of a transcriptional repressor element that regulates HPV18 late promoter activity and binds cellular proteins.

**FIG 4:**
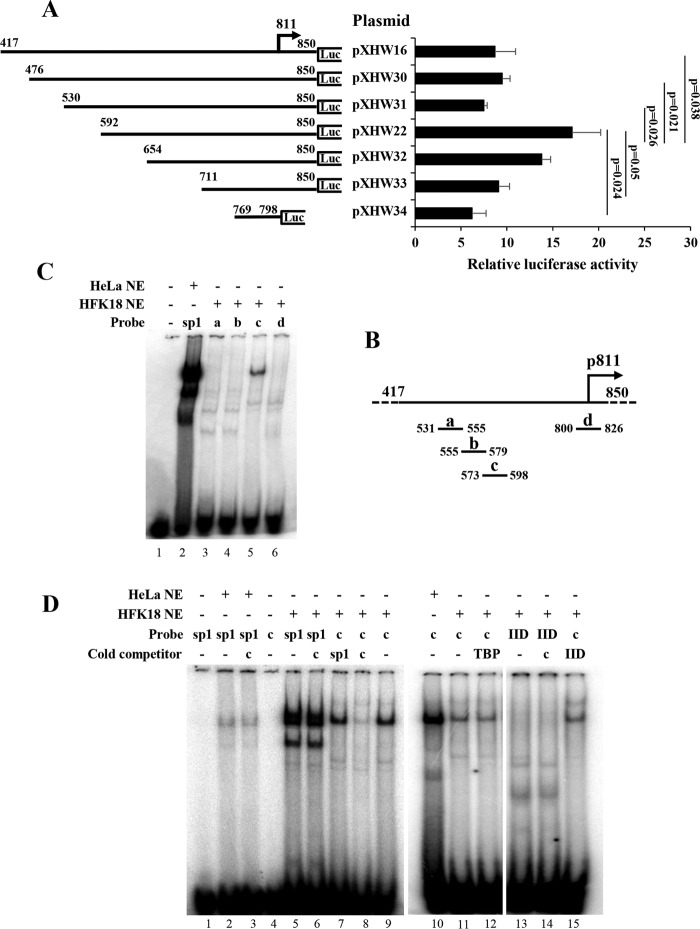
Identification of a transcriptional repressor element that affects P_811_ promoter activity and binds cellular proteins. (A) Schematic diagrams and their promoter activities for individual plasmids derived from pXHW16 with indicated deletions of the promoter region (nt 417 to nt 850 in the HPV18 genome) inserted upstream of a *firefly* luciferase (Luc) gene. The numbers above the lines represent nucleotide positions of the first and last nucleotides of the insertion in the HPV18 genome. The HPV18 late TSS at nt position 811 is indicated by an arrow. HFK18 cells in the presence of 2 mM calcium were cotransfected for 48 h with the individual plasmids along with *Renilla* luciferase plasmid pRL-TS. The supernatant of the cell lysates was examined for dual luciferase activities, and the relative promoter activity levels were calculated as described for Fig. 2A. (B) Diagrams of the nucleotide positions of four synthetic, double-stranded DNA oligonucleotide probes (a to d) used for electrophoretic mobility shift assays (EMSA). (C) Probe c, derived from nt 573 to nt 598 in the HPV18 genome, interacts with a cellular protein(s) from HFK18 cells. Probes were labeled with ^32^P, incubated with nuclear extract (NE) from HeLa or HFK18 cells, and then examined by EMSA. A Sp1 consensus oligonucleotide probe was used as a positive control. Protein-DNA complexes were resolved on a 4% native polyacrylamide gel. (D) Verification of the cellular proteins interacting with the repressor element by competitive gel shift assays. ^32^P-labeled probe c and a ^32^P-labeled Sp1 or TFIID (IID) oligonucleotide were incubated with NE prepared from HeLa or HFK18 cells in the presence or absence of an indicated cold competitor probe c, Sp1, TBP, or IID consensus oligonucleotide. Protein-DNA complexes were resolved on a 4% native polyacrylamide gel.

Given that the pXHW16 plasmid, which contains a longer version (nt 417 to 850) of the promoter region, constantly showed lower promoter activity than pXHW22 containing a short promoter version (nt 592 to 850) (Fig. 1A), we hypothesized that there might be a repressor element residing in the region from nt 417 to nt 592. To test this hypothesis, we constructed a series of successive deletions from nt 417 to 811 (Fig. 4A) and examined their effects on promoter activities. As shown in Fig. 4A, deletion of the region from nt 530 (pXHW31) to nt 592 (pXHW22) was found to increase the late promoter activity. On the basis of these results, we suspect that this region in the HPV18 genome contains a repressor element.

To examine whether the repressor element in the region of nt 530 to 592 functions by interacting with cellular proteins, we synthesized several double-stranded DNA (dsDNA) oligonucleotide probes (probes a, b, and c) spanning the entire region for protein-binding gel shift assays, along with probe d (nt 800 to 826) running over the late promoter TSS as a control (Fig. 4B). By using ^32^P-labeled, double-stranded DNA oligonucleotide probes and nuclear extract (NE) isolated from calcium (2 mM)-differentiated HFK18 keratinocytes for electrophoretic mobility shift assays (EMSA), we found that only probe c, spanning nt 573 to 598, displayed strong binding to a protein(s) similar in size to Sp1 from HeLa NE (Fig. 4C).

We next investigated whether the cellular protein(s) interacting with probe c is Sp1 or some other common transcription factors by using competitive EMSA. In the assays, HeLa or HFK18 NE derived from the cells cultured with 2 mM calcium was first incubated with gel shift binding buffer in the presence or absence of a cold competitor probe at room temperature for 10 min before addition of ^32^P-labeled probe c or Sp1. As shown in Fig. 4D (lanes 1 to 9), neither the Sp1 probe without HeLa NE nor probe c without HFK18 NE displayed any shift signal in the assay (lanes 1 and 4), but both probes showed strong binding to a protein(s) of similar size in the presence of either HeLa NE or HFK18 NE (lanes 2, 5, and 9). As shown by a DNA-binding specificity experiment, their binding was not blocked by a heterologous cold probe competitor (lanes 3, 6, and 7). Importantly, the protein interaction of HFK18 NE with probe c was blocked by a cold competitor probe, probe c (lane 8). These results indicate that the specific probe c-interacting protein(s) is not Sp1. With the same approach, we investigated the possibility that the RE-interacting cellular protein(s) might be another common transcription factor. As shown in Fig. 4D (lanes 10 to 15), neither TATA-binding protein (TBP) nor TFIID is a probe c-interacting protein, because binding of probe c with the protein(s) from HFK18 NE was not blocked either by a cold TBP competitor (compare lane 11 with lane 12) or by a cold TFIID competitor (compare lane 11 with lane 15) nor the TFIID probe binding of the NE proteins by the cold probe c competitor (compare lane 13 with lane 14).

### Identification of a repressor element core by 3-bp linker-scanning mutational analysis.

**FIG 5:**
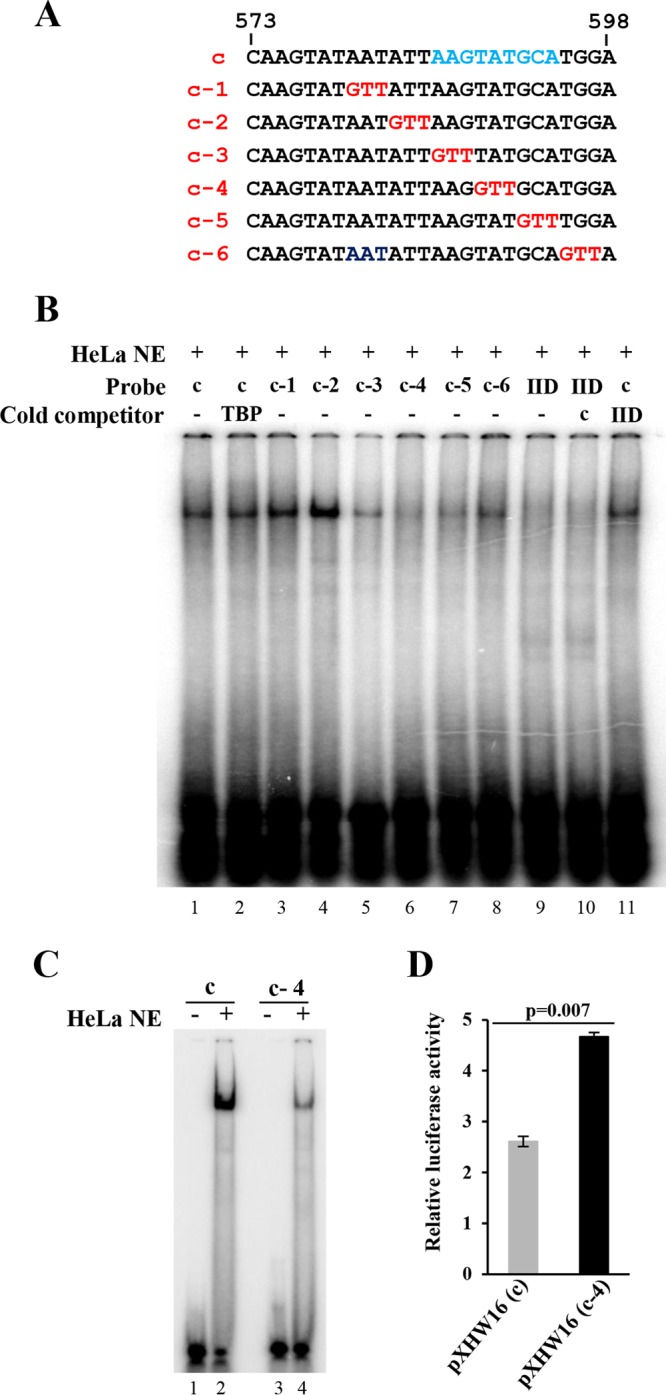
Mapping of a protein-binding core motif from the HPV18 late-promoter repressor element by 3-bp linker-scanning mutational analysis in EMSA. (A) Sequence of probe c and its substitutions with a 3-bp GTT linker. (B) Identification of an AAGTATGCA motif in the repressor element as a protein-binding core. Indicated probes were ^32^P-labeled double-stranded DNA oligonucleotide probe c and its derived variants in panel A used for EMSA with HeLa NE in the presence or absence of the indicated cold competitors. TBP and TFIID (IID) were included as controls. The protein-DNA complex was resolved on a 4% native polyacrylamide gel. (C) Protein binding profile of wild-type probe c and its c-4 mutant in EMSA. (D) Replacement of the probe c-corresponding sequence in plasmid pXHW16 with a c-4 sequence promotes HPV18 late promoter activity in HFK18 cells.

To delineate the RE core sequence in probe c for interaction with a cellular protein(s), a 3-base mutation, GTT, was introduced progressively from 5′ to 3′ to scan the 26-bp sequence (Fig. 5A). Each dsDNA oligonucleotide with the mutation was analyzed and compared with the wild-type (wt) c probe by EMSA using HeLa NE, which also contains such a protein(s) (Fig. 4D, lane 10). As shown in Fig. 5B, probes c-1, c-2, and c-6 with the indicated mutation (Fig. 5A) did not affect the protein binding (lanes 3 to 4 and 8), but probes c-3, c-4, and c-5 (lanes 5 to 7) did do so. These observations suggest the 9-nt sequence AAGTATGCA to be the core sequence of the protein-interacting repressor element. Competitive EMSA also indicated the absence of competitive binding to probe c by TBP and TFIID binding motifs (Fig. 5B, lanes 2, 10, and 11). The distinctive change of protein binding from the wt c probe to the mutated c-4 probe was further supported by a remarkable reduction in the level of unbound wild-type probe c (Fig. 5C). We also compared the promoter activities in pXHW16 with a wild-type repressor element core sequence and in the derived pXHW47 plasmid containing a c-4 mutation sequence in the promoter region. As shown in Fig. 5D, introduction of mutation c-4, which has the weakest protein binding activity, into the pXHW16 repressor element core led to increased promoter activity.

### Presence of an active repressor element-binding protein(s) only in differentiated HFK18 keratinocytes.

**FIG 6:**
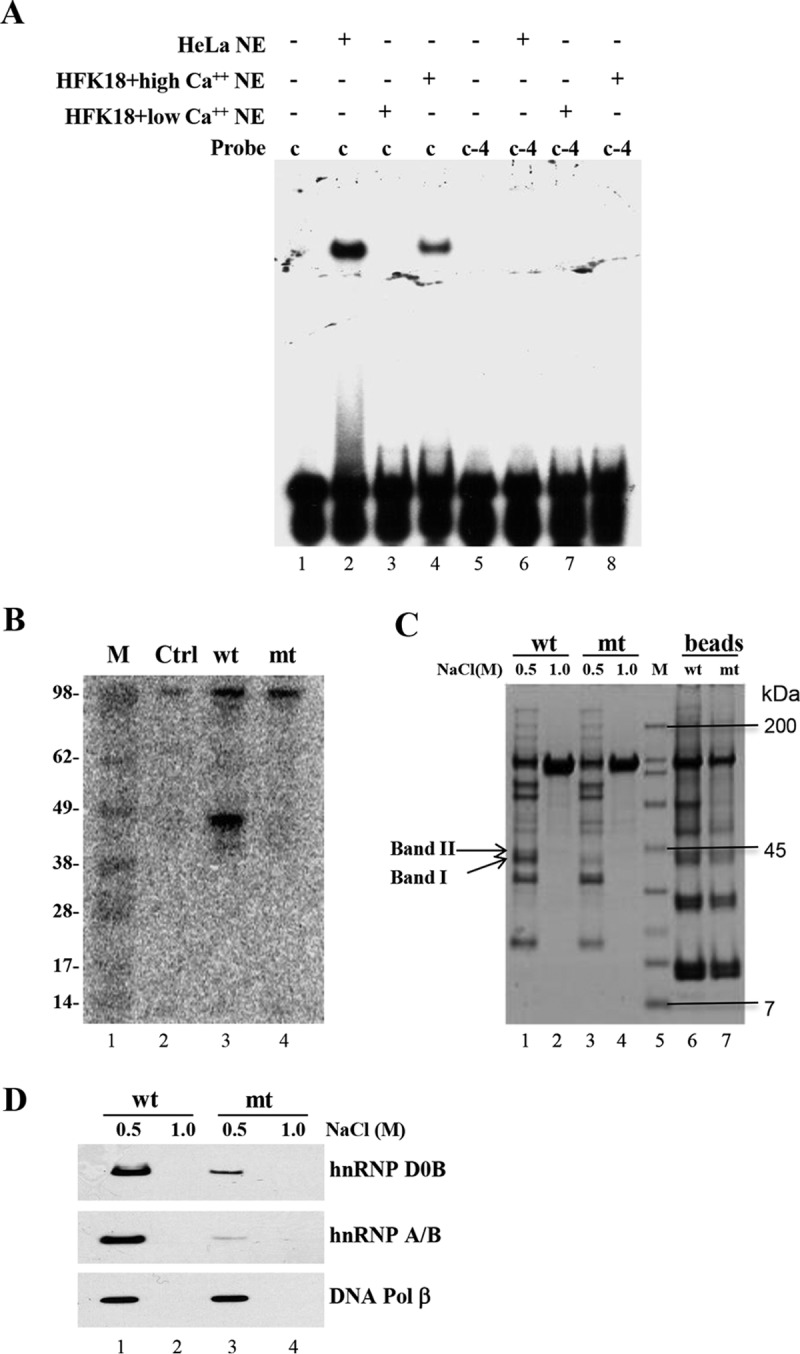
Identification of the cellular protein(s) bound to the repressor core element and its relation to HPV18 late promoter activity. (A) Expression of the repressor element-binding protein(s) in HFK18 cells depends on cell differentiation. ^32^P-labeled, double-stranded DNA probe c and its c-4 mutant probe used for EMSA were incubated with NE of HFK18 cells cultured under low- or high-calcium conditions. HeLa NE served as a control. (B) The repressor element-binding protein(s) (~45 kDa) is detectable by Southwestern blotting. Biotin-labeled dsDNA oligonucleotide probe c (wt) and its c-4 mutant (mt) were used for protein pulldown assays. The proteins pulled down from HeLa NE were separated by SDS-PAGE, transferred onto a nitrocellulose membrane, renatured, and probed by a ^32^P-labeled double-stranded DNA oligonucleotide probe (c). Ctrl, control. (C) Isolation of the repressor-binding protein(s) for LC-MS/MS analysis. Biotinylated probe c (wt) or its c-4 mutant (mt) was immobilized on magnetic streptavidin beads and incubated with HeLa NE. After extensively washing, bound proteins on the beads were eluted with a buffer containing 0.5 M or 1.0 M NaCl. Eluted fractions and the eluted beads were analyzed by SDS-PAGE and visualized by Coomassie blue staining. The specific repressor element-binding proteins (indicated with arrows) corresponding to probe c were excised for LC-MS/MS analysis. M (lane 5), Bio-Rad broad-range protein markers (7, 14, 21, 31, 45, 66, 97, 116, and 200 kDa). (D) Verification of repressor element-binding proteins hnRNP D0B and hnRNP A/B from the bound proteins on the beads by Western blotting using corresponding antibodies. Pol, polymerase.

It has been well documented that both vegetative DNA replication and late promoter transcription of BPV-1 and other HPV genotypes depend on keratinocyte differentiation. In particular, levels of viral late transcripts encoding the L1 and L2 capsid proteins increase dramatically upon keratinocyte differentiation (8, 56). To determine whether the identified repressor element core-binding protein(s) in HFK18 cells depends on cell differentiation, we examined the binding activity of the repressor element-binding protein(s) in low (0.5 mM)- or high (2.0 mM)-calcium-treated HFK18 cells by EMSA, with HeLa NE serving as a control because HeLa cells were constantly cultured under high-calcium conditions. As shown in Fig. 6A, the probe c-binding activity could be detected only in high-calcium-treated HFK18 cells and in HeLa cells, whereas the c-4 mutant (mt) probe did not show any binding activity in any of the tests. Data suggest that the binding activity of the repressor element-binding protein(s) is increased following keratinocyte differentiation, presumably to provide counteracting regulatory properties in the late transcription *in vivo*.

### Isolation and characterization of the cellular proteins binding to the repressor element core.

To identify the repressor element-binding protein(s), we first performed Southwestern blotting to investigate the size of the repressor element-interacting protein(s) in HeLa NE. The proteins pulled down from biotin-labeled probe c (oXHW181 plus oXHW198) or the c-4 mutant probe (oXHW191 plus oXHW199) were first separated by SDS-PAGE and transferred to a nitrocellulose membrane. The membrane-bound proteins were renatured and then incubated with ^32^P-labeled probe c (oXHW180 plus oXHW181). As shown in Fig. 6B, Southwestern blotting detected a protein(s) of ~45 kDa binding to probe c (wt) but not to mutant probe c-4 (mt).

We next purified the repressor element-binding protein(s) from HeLa NE by a large-scale pulldown assay with probe c (oXHW181 plus oXHW198)-immobilized streptavidin magnetic beads. Mutant probe c-4 served as a negative control. The proteins pulled down by probe c and probe c-4 were separated by NuPAGE gel electrophoresis and visualized by Coomassie blue staining (Fig. 6C), and the probe c-specific proteins of ~45 kDa (Fig. 6C, arrows) were excised for protein identification by mass spectrometry. By this analysis, two major candidate proteins specific for the probe c binding were identified to be hnRNP D0B/AUF1 (4 alternative spliced isoforms: p37, p40, p42; and p45) (57–61) and hnRNP A/B (62–64) (see Table S1 in the supplemental material). Other factors related to DNA replication/repair, such as DNA polymerase β, aprataxin, replication factors C2 (RFC2), C3 (RFC3), and C4 (RFC4), and hnRNPC1/C2, were also identified as minor binding factors (Table S1). The more efficient binding of hnRNP D0B and hnRNP A/B to probe c than to mt c-4 was further verified by Western blotting by using the corresponding antibodies (Fig. 6D). As a control, the binding of DNA polymerase β to probe c was analyzed and found to be similar to the binding of mt c-4 (Fig. 6D).

10.1128/mBio.00713-17.5 TABLE S1  Peptides identified by LC-MS/MS analysis. Download TABLE S1, PDF file, 0.02 MB. Copyright © 2017 Wang et al. 2017 Wang et al. This content is distributed under the terms of the Creative Commons Attribution 4.0 International license.

**FIG 7:**
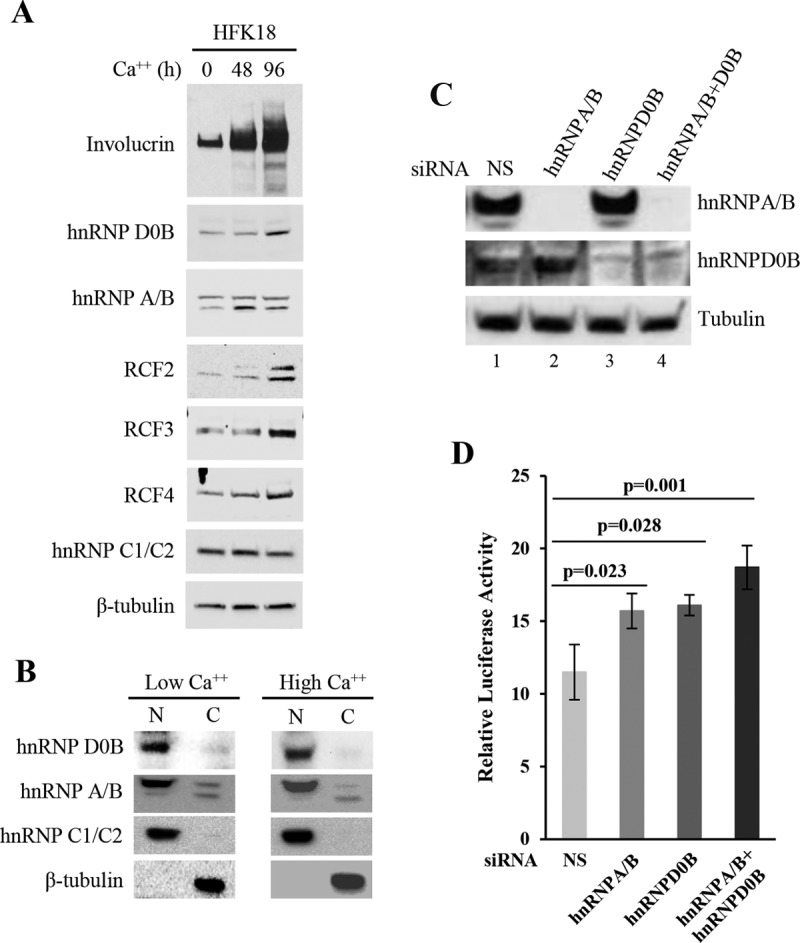
Expression and function of hnRNP D0B and hnRNP A/B in HFK18 keratinocytes. (A) Expression of hnRNP D0B, hnRNP A/B, RFC2, RFC3, and RFC4 in HFK18 cells under conditions of calcium-mediated differentiation. HFK18 cells were incubated with calcium-free EpiLife medium (no FBS) supplemented with 1× HKGS for 24 h, followed by induction of cell differentiation for 48 or 96 h with 3 mM Ca^2+^ EpiLife medium (5% FBS, 1× HKGS, 3 mM CaCl_2_) before total protein sample preparation was performed for Western blotting of individual proteins with corresponding antibodies. Involucrin served as a keratinocyte differentiation marker and β-tubulin and hnRNP C1/C2 as loading controls. (B) Nuclear and cytoplasmic distribution of hnRNP D0B and hnRNP A/B by cell fractionation of HFK18 cells under low- or high-calcium conditions. See details in the panel A legend. (C and D) Knockdown of hnRNP D0B and hnRNP A/B expression in HFK18 cells promotes HPV18 late promoter activity. The cells with efficient knockdown of the corresponding protein (C) were cotransfected by plasmid pXHW16 and pRL-TS and examined for HPV18 late promoter activity in the presence of 3 mM calcium (D). Nonspecific (NS) siRNA served as a control. hnRNP A/B and hnRNP D0B siRNA knockdown efficiency in HFK18 cells (C) was examined by Western blotting.

Further investigations showed increased levels of expression of hnRNP D0B, hnRNP A/B, RFC2, RFC3, and RFC4 and isoform production of hnRNP A/B and RFC2, along with increased expression of the keratinocyte differentiation marker involucrin in HFK18 cells following calcium-induced differentiation (Fig. 7A). Fractionation of HFK18 cells grown under low- or high-calcium conditions showed that the hnRNP D0B and hnRNP A/B that were identified are found mainly in the nucleus (Fig. 7B), although a small amount of hnRNP A/B could be found in the cytoplasm, with a proportional increase of the smaller isoform of hnRNP A/B under the high-calcium conditions (Fig. 7B). To correlate the expression of hnRNP D0B and hnRNP A/B and the late promoter activity, we knocked down the expression of hnRNP D0B or hnRNP A/B in HFK18 cells and examined the knockdown effect on HPV18 late promoter activity of the pXHW16 plasmid bearing the repressor element in its promoter. As shown in Fig. 7C and D, specific small interfering RNA (siRNA) knockdown of hnRNP D0B or hnRNP A/B in HFK18 cells promoted HPV18 late promoter activity. Double knockdown of hnRNP D0B and hnRNP A/B led to an even higher level of activity. In contrast, knocking down of the same factors did not enhance the promoter activity in pXHW47 containing the c-4 mutation (data not shown).

### Identification of HPV18 late promoter-associated small RNAs.

Recent studies revealed the coexistence of promoter-associated small RNAs (PASRs) or transcription initiation RNAs (tiRNAs) from both DNA strands directly adjacent to the TSS (65, 66). Subsequently, we screened for the possible existence of these small RNAs in day 8, 10, 12, and 16 raft culture tissues derived from primary human keratinocytes productively infected by HPV18 using small RNA-sequencing (RNA-Seq). As shown in Fig. S4, there were several small RNA-enriched peaks in HPV18-infected raft tissues compared to uninfected raft tissues derived from primary human keratinocytes. One small-RNA peak is shown downstream of the early promoter P_102_ TSS from nt 217 to 234, and three are shown adjacent to the late promoter P_811_ TSS. These HPV18 small RNAs were sense strand RNAs and had a size of ~16 to 25 nt (Fig. S4A). They are not microRNAs (miRNAs) and lack the characteristic uniform 5′ end and stem structures of miRNA. By focusing on the HPV18 genome position from nt 759 to nt 1009 (Fig. S4B), we found that two peaks around the HPV18 late promoter P_811_ TSS (nt 810 to nt 873) are ~14 nt apart and are more abundant in day 12 and day 10 rafts. Moreover, the peak around nt 911 to nt 929 is located immediately upstream of the nt 929 5′ splice site. Interestingly, the peak from nt 217 to nt 234 is also immediately upstream of the nt 233 5′ splice site, and it was the highest for the day 10 rafts followed by the day 12 rafts. These small RNAs could represent splice-site small RNAs (spliRNAs) (67, 68). Together, our results demonstrated for the first time the presence of HPV18 late promoter-associated, transcription-related tiRNAs/PASRs and splicing-associated spliRNAs during productive HPV18 infection.

10.1128/mBio.00713-17.4 FIG S4  Identification of HPV18 late promoter-associated small RNAs by small RNA-Seq. (A) Annotation of HPV18-specific small RNAs over the viral infection time (days) against the HPV18 genome. Expression of HPV18-specific small RNAs in day 8, 10, 12, and 16 HFK rafts with or without HPV18 infection were examined by small RNA-Seq. (B) Close look at the read peaks of the annotated viral small RNAs in the late promoter TSS region shown in panel A. The read peaks from different infection times (days) are colored; arrows indicate the common TSS p811 at the HPV18 late-promoter region and a 5′ splice site (5′ ss) at nt position 929 often used for splicing of both viral early and late transcripts. Download FIG S4, PDF file, 0.1 MB. Copyright © 2017 Wang et al. 2017 Wang et al. This content is distributed under the terms of the Creative Commons Attribution 4.0 International license.

## DISCUSSION

### HPV late promoter, viral DNA replication, and DNA strand-bias effect.

It has been known for decades that the activity of viral late promoters is associated with viral DNA replication among almost all DNA viruses (13–15, 17–22, 69–73) and even dsDNA bacteriophage N4 (and T4) (23, 24). Inhibition of DNA replication by phosphonoacetic acid (PAA) or ganciclovir (GCV) (19, 21) decreases viral late promoter activity. However, the mechanism of how DNA replication activates the late promoter and what components of the replication machinery are involved remain largely unknown. Recent comparative and evolutionary analyses of the genome sequences from different species indicated that the archaeal promoters and replication origins have similar sequence compositions and are the last universal common cellular ancestors (74) and that the replication origins associated with gene promoters are conserved in mouse and vole X chromosome (75). For this report, we mapped HPV18 late promoter to a TATA-less region in the E7 ORF and demonstrated that activation of an HPV18 late promoter depends on the origin of HPV18 DNA replication being oriented in one direction relative to the late promoter in HFK18 cells. Our study confirmed this DNA strand-bias effect on viral late promoter activity by using the DNA polymerase inhibitor aphidicolin (52) and by transfection of single-strand oligonucleotides in a manner similar to procedures described before (53–55).

A well-known precedent exists where the direction of replication controls the activity/switchability of the mating-type locus (*mat1*) of fission yeast cells (76) and where the orientation of DNA replication establishes the mating-type switching pattern in *Schizosaccharomyces pombe* (76). Polar replication termination site 1 (RTS1) blocks *mat1* replication in one direction, ensuring that *mat1* is always unidirectionally replicated in the chromosome. The *mat1* switching does not occur at all when it is inverted in the chromosome or when the RTS1 element is moved to the other side of the *mat1* locus (77). Thus, a DNA replication-arrest RTS1 site regulates imprinting by determining the direction of replication at *mat1* in *S. pombe* (77). Other precedents described for bacteriophage T4 replication direction and late transcription include the demonstration that sliding-clamp processivity factor gp45 of the T4 DNA polymerase functions as an activator of T4 late transcription (25, 78–80). Similarly, our results presented here show that the late promoter activity is modulated by the direction of HPV18 DNA replication.

The precise mode of papillomavirus DNA replication has been a topic of debate. It is thought that HPV16 and HPV31 DNA replication in undifferentiated cells occurs primarily by means of theta structures and in a bidirectional manner. However, the replication changes to rolling-circle, asymmetric, and unidirectional amplification when the cells are grown under differentiation conditions (32) or when replication is reconstituted in epithelial cell extract (33). Rolling-circle replication has been observed possibly to occur at a low level in HPV11 (36) and also as a mechanism for BPV-1 DNA replication (34, 35). A recent report indicates that HPV genomes in an osteosarcoma cell line exert an alternative, recombination-dependent replication mechanism (37, 38). When the pXHW15 and pXHW21 plasmids were replicated by Ori in the CW orientation, we expected both plasmids to be efficient with respect to its activation of HPV18 late promoter, as the Ori of each is oriented with respect to the late promoter, just as is the case in the virus genome. However, the promoter in pXHW15 or pXHW21 was nearly inactive in the differentiated HFK18 cells, suggesting some inhibitory effect of replicating foreign plasmid DNA. This could have been the effect of blocking the normal switching to the unidirectional replication mode. In HPV11, Auborn and colleagues found replicative DNA intermediates containing single forks with a mass greater than half of the replicated molecules (36). We suggest that oligonucleotides may allow the replication to ensue unidirectionally in the opposite direction by annealing oligonucleotides to the DNA lagging strand being replicated from this Ori and that priming by either oligonucleotide may activate the late promoter (Fig. 3).

Plasmids pXHW16 and pXHW22 with the CCW Ori, on the other hand, exhibit an active promoter which is sensitive to the alphidicolin DNA replication inhibitor (52). This Ori effect may be a consequence of the fact that it is already replicating unidirectionally. Although more-explicit information concerning the mechanism of how the direction of replication activates the late promoter remains to be determined, our data suggest that a replication fork moving toward the late promoter in one direction may be essential for activation. Moreover, our results indicate that viral DNA replication follows a mechanism of activation of the HPV18 late promoter different from that of the KSHV K8.1 late promoter, which requires the viral OriLyt-L and does not depend upon the OriLyt-L orientation (21). One interpretation of the different strategies employed could be that papillomaviruses transcribe viral genes from only one strand of the genome DNA whereas KSHV transcribes from both strands.

### HPV18 late promoter’s repressor element and repressor element-binding proteins.

Proteins and other factors have been found to regulate DNA replication-dependent late promoter activity. Early studies on adenovirus major late promoter (MLP) identified a 40-kDa protein binding to the TTGTCAGTTT motif within a region downstream of the promoter. Binding occurs only after the onset of viral DNA replication (71). Ken Matsumoto and colleagues identified an adenovirus template activating factor, adenovirus template activating factor-I (TAF-I), from uninfected HeLa cytoplasmic fractions that promotes the formation of preinitiation replication complexes and also MLP promoter activity in cell extracts (72). Moreover, knockdown of transcriptional repressor CTCF (CCCTC-binding factor) was found to suppress viral DNA replication as well as late, but not early, gene expression. Binding of CTCF to viral chromatin depends on viral DNA replication (73). In our study, we identified a repressor element (5′-AAGTATGCA-3′) in the HPV18 late promoter that modulates late promoter activity through its interaction with repressor element-binding proteins. These repressor element-binding proteins exhibited DNA-binding activity in calcium-differentiated HFK18 nuclear extract and are nuclear hnRNP D0B (also named AUF1) and hnRNP A/B. Regardless of their possible modifications, these proteins are differentially expressed during HFK18 differentiation. Both proteins are RNA- and DNA-binding proteins and could function as transcription factors or repressors for mammalian promoters (60–63, 81, 82) and for an EBV C promoter (83). Consistent with these findings (62, 63), our data suggest that the binding of hnRNP D0B and hnRNP A/B to the HPV18 late promoter may regulate the level of the DNA replication-dependent promoter. However, more studies are needed to understand (i) whether these proteins also modulate viral DNA replication, (ii) whether their DNA binding activities are coupled to protein modifications in the presence of calcium, and (iii) whether other unknown proteins or regulatory *cis* elements contribute to regulation of HPV18 late promoter activity. For comparison, we note that adenovirus late IVa2 transcription is regulated by viral DNA synthesis-dependent relief of transcriptional repression by a cellular IVa2-RF protein (15).

### HPV18 late transcription and production of tiRNAs and spliRNAs.

TiRNAs or PASRs that are ~18 nt in length are derived from the region consisting of nt −60 to nt +120, including TSS from the same TSS strand (65, 66). SpliRNAs are nucleus-specific ~17- to 18-nt small RNAs whose 3′ ends map precisely to the splice donor site of internal exons in animals (67). Both tiRNAs and spliRNAs are associated with highly expressed genes. In the present study, we demonstrated for the first time the presence of HPV18 late promoter-associated or transcription-related tiRNAs and 5′ splice donor site-associated spliRNAs derived from the nt 233 and the nt 929 splice donor sites in the HPV18 genome, which were highly associated with productive HPV18 infection. Although the function of the tiRNAs remains unknown, recent reports suggest that they may play a role in nucleosome positioning (66), chromatin organization (84), or RNA-directed transcriptional gene silencing (85). By analogy, we postulate that the viral tiRNAs identified in association with HPV18 late promoter activation may regulate the HPV18 late promoter activity and gene expression. Because the small-RNA peak in the region extending from nt 217 to nt 234 has a 3′ end largely mapped to the nt 233 splice donor site, a region further downstream of the viral early transcription start site at nt 102, we view this species as a spliRNA peak rather than as a tiRNA peak. However, the identified spliRNAs associated with the viral 5′ splice donor sites could be the by-products of viral pre-mRNA splicing. This presumption appears to be supported by the observation that more spliRNAs were derived from the nt 929 splice donor site than from the nt 233 splice donor site because the nt 929 splice donor site is a common splice donor for splicing of both viral early and viral late RNAs whereas the nt 233 splice donor site in the E6 ORF is used only for splicing of viral early RNAs. Whether such spliRNAs play a role in regulation of RNA splicing or in formation of the exon junction complex (EJC) (86) to promote RNA export and translation remains to be investigated.

## MATERIALS AND METHODS

### Plasmid construction.

All plasmids described in this report were derived from a *firefly* luciferase reporter pGL3 basic vector with insertion of an AgeI-NsiI linker at the vector BamHI site (Fig. 1). Plasmids pXHW15, pXHW16, pXHW17, pXHW18, pXHW19, pXHW20, pXHW21, and pXHW22 were constructed by inserting a 124-bp segment of the HPV18 Ori (nt 7805 to nt 7857 and nt 1 to nt 72) (44, 45) in a plasmid-relevant counterclockwise (CCW) or clockwise (CW) orientation between the AgeI and NsiI sites downstream of the Luc ORF in the modified pGL3-luciferase reporter vector. The expression of Luc in these vectors is controlled by a long (nt 417 to nt 850) or short (nt 592 to nt 850) HPV18 late promoter in sense (S) or antisense (AS) orientation relative to the *luc* gene at the Asp718 and XhoI sites as described for Fig. 1A and B as well as for Fig. S1 in the supplemental material. Note that the CW and CCW orientation of Ori describes the Ori sequence orientation of the clone in the plasmid and not the direction of replication itself.

See Table S2 in the supplemental material for details of the individual primer sequences used for amplification of a PCR insertion of HPV18 Ori or an HPV18 late-promoter region from an HPV18 plasmid as follows: oXHW123 (forward [F]) and oXHW124 (backward [B]) for the Ori insertion in CW orientation; oXHW125 (F) and oXHW126 (B) for the Ori insertion in CCW orientation; oXHW127 (F) and oXHW128 (B) for the promoter (nt 417 to nt 850) in sense (S) orientation; oXHW129 (F) and oXHW130 (B) for the promoter (nt 417 to nt 850) in antisense orientation (AS); oXHW131 (F) and oXHW128 (B) for the promoter (nt 592 to nt 850) in S orientation; and oXHW132 (F) and oXHW130 (B) for the promoter (nt 592 to nt 850) in AS orientation. Plasmid pXHW28 has the same HPV18 promoter as pXHW22 but lacks the HPV18 Ori, and pXHW61 was derived from pXHW21 but has its Ori changed to CCW orientation. Plasmids pXHW30, pXHW31, pXHW32, and pXHW33 were all derived from plasmid pXHW16 by replacing its putative HPV18 late promoter at the Asp718 and XhoI sites with a shorter version of the promoter amplified from HPV18 plasmid DNA by a primer pair consisting of oXHW170 (F), oXHW171 (F), oXHW172 (F), or oXHW173 (F) and oXHW128 (B). Plasmid pXHW34 was derived from pXHW22 by replacing its promoter at the Asp718 and XhoI sites with a short, double-stranded DNA fragment (annealed oXHW174 and oXHW175 oligonucleotides) corresponding to the region consisting of nt 769 to nt 798 in the HPV18 genome. Plasmid pXHW47 was derived from pXHW16 and has a mutated repressor core (c-4)-binding site at the region consisting of nt 573 to nt 598 in its promoter. An overlapping PCR strategy (87) was used to introduce the point mutations in which a PCR product generated from HPV18 DNA with primer pair oXHW127 (F) and oXHW191 (B) was annealed with a PCR product generated from HPV18 DNA with oXHW190 (F) and oXHW128 (B), reamplified by primer pair oXHW127 and oXHW128, and then swapped into plasmid pXHW16 at Asp718 plus XhoI sites. Both plasmid pXHW49 and plasmid pXHW50 have an SV40 early promoter derived from a pGL3 control vector (Promega) and an HPV18 Ori. However, pXHW49 was derived from pXHW21 and has the Ori in CW orientation and pXHW50 was derived from pXHW22 and has the Ori in CCW orientation (Fig. S1C). The SV40 early promoter was generated by PCR from the pGL3 control vector with primer pair oXHW236 (SV40 promoter nt 48 to nt 69) and oXHW237 (SV40 promoter nt 275 to nt 258) and was swapped into pXHW21 or pXHW22 at the Asp718 plus XhoI sites. Plasmids pMA102, pMA103, pHBL10, and pHBL11 were all derived from pXHW28. The pMA102 has a CW Ori and pMA103 has a CCW Ori inserted immediately upstream of the late promoter at the Asp718 site. Plasmid pHBL10 was also derived from pXHW28 but contains a CW Ori, and its downstream sequences (HPV18 nt 73 to nt 591) are separated by a synthetic poly(A) signal from the HPV18 late promoter. Plasmid pHBL11 was derived from pHBL10 but has a CCW Ori (Fig. S1D; Table S2).

10.1128/mBio.00713-17.6 TABLE S2  Oligonucleotide primers and oligonucleotides used in the study. Download TABLE S2, DOCX file, 0.02 MB. Copyright © 2017 Wang et al. 2017 Wang et al. This content is distributed under the terms of the Creative Commons Attribution 4.0 International license.

### HPV18-immortalized foreskin keratinocyte (HFK18) differentiation, transfection, and dual-luciferase assay.

HPV18-immortalized karytinocytes (HFK18) derived from primary foreskin keratinocytes were cultured in complete EpiLife medium (Invitrogen) (calcium free) supplemented with 1% human keratinocyte growth supplement (HKGS) and 5% fetal bovine serum (FBS) before use for transfection or for differentiation induction with a high concentration (2 mM) of calcium. HFK18 cells contain an episomal HPV18 genome which expresses viral E1 and E2 by reverse transcriptase PCR (RT-PCR) (Fig. S3) and can be induced for viral DNA replication and production of virions in raft culture (88, 89).

In general, mitomycin C-treated 3T3 cells (J2 feeder cells) were prepared at 24 h before keratinocyte transfection and seeded into a 24-well plate at 0.4 × 10^5^ cells per well. After the 3T3 cells had attached, HFK18 cells were then seeded at 1 × 10^5^ cells per well on the feeder cells and refed on the following day with 0.5 ml/well of fresh EpiLife medium (calcium free) or F12 medium (calcium free) supplemented with 5% FBS, 1% HKGS, and 2 mM calcium for 30 to 60 min before transfection. The cells were then cotransfected for 48 h with 600 ng of the testing *firefly* luciferase reporter plasmid together with 300 ng of *Renilla* luciferase plasmid pRL-TS (21) using LipoD 293 DNA *in vitro* transfection reagent (SignaGen Laboratories, Ijamsville, MD) according to the manufacturer’s instructions. The supernatant of the cell lysates was examined for dual luciferase activities using a dual-luciferase reporter assay system (Promega). Relative luciferase activity was calculated by dividing the value representing the light unit readings obtained from a *firefly* luciferase reporter construct by the value representing the light unit readings obtained from the *Renilla* luciferase control in this cell reporter.

To examine DNA replication-mediated HPV18 late promoter activity, HFK18 cells (1 × 10^5^ cells per well in a 24-well plate after attachment) were cultured in F12 medium (calcium free) with aphidicolin overnight at a dose of 0, 3, or 6 µM followed by changing to F12 medium with the same amount of aphidicolin and 2 mM calcium 60 min before plasmid transfection. The cells cultured in the F12 medium with aphidicolin (Sigma) were transfected for 24 h with 600 ng of the testing *firefly* luciferase reporter pXHW22 plasmid together with 100 ng of a *Renilla* luciferase pRL-SV40 plasmid (Promega) using LipoD transfection reagent, and the supernatant of the cell lysate was examined for dual luciferase activities as described above.

### HFK18 cell nuclear extract preparation and fractionation.

HFK18 cells were grown in complete, calcium-free EpiLife medium and treated with or without 2 mM calcium for 48 h before they were subjected to nuclear extraction. The cells were then washed twice with phosphate-buffered saline (PBS), trypsinized, and resuspended in 10 ml of the complete EpiLife medium. After spinning down was performed, the cell pellet was resuspended and washed 3 times with ice-cold PBS and then resuspended in 0.4 ml of buffer A (10 mM HEPES [pH 7.9], 1.5 mM MgCl_2_, 10 mM KCI, 1 mM dithiothreitol [DTT], 1 mM phenylmethylsulfomyl fluoride), incubated for 15 min on ice, and homogenized with a Dounce homogenizer B pestle for about 20 strokes. The homogenized cells were then centrifuged at 14,000 rpm for 15 min at 4°C. The nuclear pellet was resuspended in 0.2 ml of buffer B (20 mM HEPES [pH 7.9], 25% [vol/vol] glycerol, 0.42 M NaCl, 1.5 mM MgCl_2_, 0.2 mM EDTA, 1 mM DTT, 1 mM phenylmethylsulfonyl fluoride), incubated on ice for 30 min, and then centrifuged at 14,000 rpm for 20 min at 4°C. Supernatants were collected, divided into aliquots, and stored at 70°C.

Nuclear and cytoplasmic fractionation of HFK18 cells was performed using a nuclei isolation kit from Sigma (catalog no. NUC101) according to the protocol provided by the company, with slight modification. Briefly, cells growing in a 60-mm-diameter dish were scraped in 1× PBS and lysed using 400 µl Nuclei EZ lysis buffer. Nuclei were pelleted by centrifugation (500 × *g*) for 5 min at 4°C. The supernatant was collected as the cytoplasmic fraction, and the obtained nuclear pellet was resuspended and washed gently one time with 1× PBS and then resuspended in 400 µl lysis buffer. Equal volumes of nuclear and cytoplasmic extracts or total protein samples were examined by a standard Western blot protocol after solubilization in Laemmli SDS buffer supplemented with 5% (vol/vol) 2-mercaptoethanol (2-ME).

### Electrophoretic mobility shift assay (EMSA).

EMSA was performed using a gel shift assay system (Promega). Nuclear protein extracts from HFK18 cells were prepared as described above. HeLa nuclear extract was included in the gel shift assay system as a positive control. The double-stranded oligonucleotides used as probes were 5′ end labeled with [γ-^32^]pATP by T4 polynucleotide kinase and purified using Illustra MicroSpin G-25 columns (GE Healthcare). The oligonucleotides used as probes and as cold competitors are shown in Table S2. Nuclear extracts were incubated with gel shift binding buffer in the presence or absence of a cold competitor oligonucleotide and incubated at room temperature for 10 min, and then ^32^P-labeled double-stranded oligonucleotide probes were added for an additional 20 min of incubation at room temperature. After 1 µl of room-temperature gel loading, 10× buffer was added per reaction and the reaction mixture was electrophoresed through a 4% polyacrylamide gel in 0.5× Tris-borate-EDTA (TBE) buffer, first at 200 V for 10 min and then at 350 V for an additional 50 min. The gel was then dried and exposed to a PhophorImager screen. The image was captured using a Molecular Dynamic PhosphorImager Storm 860 system and analyzed with ImageQuant software.

### Southwestern blotting analysis.

Five microliters of a DNA oligonucleotide, oXHW181 (20 µM), containing the specific sequence of the repressor core element was annealed with 5 µl of a biotin-labeled oligonucleotide, oXHW198 (20 µM). Similarly, 5 µl of a DNA oligonucleotide, oXHW191 (20 µM), containing the mutated c-4 sequence of the repressor core element was annealed with 5 µl of a biotin-labeled oligonucleotide, oXHW199 (20 µM), in a 10-µl reaction mixture. The annealed DNA oligonucleotides were then added to prewashed NeutrAvidin agarose resins in 100 µl of 2× binding buffer (20 mM Tris [pH 7.5], 200 mM NaCl, 6 mM EDTA, protease inhibitor cocktail tablet), and 200 µl of 1× binding buffer was added to the reaction mixture. After 30 min of incubation at room temperature with rotation followed by three washes performed with 1× binding buffer, 5 µl (~40 µg) of HeLa nuclear extract (Protein One) was added to each reaction tube and incubated for 1 h at room temperature with rotation. After three washes with 1× binding buffer, 40 µl of 2× SDS sample buffer containing 5% 2-mercaptoethanol was added to each reaction tube and then denatured by heating at 95°C for 10 min and separated in a NuPAGE 4% to 12% bis-Tris gel in 1× NuPAGE MOPS (morpholinepropanesulfonic acid) SDS running buffer. After transfer, proteins on the nitrocellulose membrane were renatured in 5 ml TNED buffer (10 mM Tris [pH 7.5], 50 mM NaCl, 0.1 mM EDTA, 1 mM DTT) containing 5% skim milk overnight at room temperature with shaking under conditions of protection from exposure to light. The buffer in the membrane container was replaced with 3 ml TNED buffer containing 5% skim milk. After addition of 20 pmol of ^32^P-labeled oligonucleotide probes (oXHW180 plus oXHW181) containing the repressor core element, the membrane was shaken in the TNED buffer overnight at room temperature under light-tight conditions, washed three times (for 15 min each time) with TNED buffer at room temperature, and then exposed to a PhophorImager screen. The image was captured using a Molecular Dynamic PhosphorImager Storm 860 system and analyzed with ImageQuant software.

### Replicated plasmid isolation from HFK18 cells and DpnI/MboI digestion analysis.

HFK18 cells were cultured in complete EpiLife medium (calcium free). At one day before transfection, HFK18 cells were seeded into seven 10-cm-diameter dishes at 8 × 10^6^ cells per dish per plasmid and, on the following day, the medium was replaced with 3 ml of fresh, complete EpiLife medium supplemented with 2 mM calcium for 30 to 60 min before transfection. The cells were then transfected with pXHW21 or pXHW22 (at 24 µg/dish) using Lipofectamine LTX and Plus reagent (Invitrogen) according to the manufacturer’s instructions. In brief, 24 µg of plasmid DNA was diluted in 3 ml of Opti-MEM I medium for 5 min at room temperature and then Lipofectamine LTX and Plus reagent were directly added to the diluted DNA. After 30 min of incubation at room temperature, A DNA-lipid complex was then added to the cells and incubated for 48 h at 37°C. Replicated plasmid DNA was extracted from transfected HFK18 cells using a QIAprep Spin Miniprep kit (Qiagen), extracting only replicated plasmid DNA. Briefly, the cells were washed once with PBS and then trypsinized and resuspended in the complete EpiLife medium. After centrifugation, the cell pellets were washed with PBS and resuspended in 250 µl of buffer P1 (Qiagen) and then lysed by adding 250 µl of buffer P2 (Qiagen). After 5 min of incubation at room temperature, genomic DNA and cell debris were precipitated by addition of 350 µl of buffer N3 (Qiagen), incubation on ice for 5 min, and then centrifugation at 10,000 × *g* for 10 min. The supernatant was loaded onto a QIAprep spin column and centrifuged for 1 min at 10,000 × *g*, followed by one wash with 500 µl of buffer PB (Qiagen) to remove residual endonuclease and then one wash with 750 µl of buffer PE (Qiagen) to remove residual salts. Plasmid DNA was then eluted in 100 µl Tris-EDTA (TE) buffer by incubation for 5 min at 37°C and centrifuged for 1 min at 10,000 × *g*. The eluted plasmid DNA was precipitated, air-dried, dissolved in water, and then digested with DpnI and/or MboI. The digested plasmid DNA was then resolved by the use of a 1% agarose gel and imaged by ethidium bromide staining.

### Purification and identification of the late-promoter repressor core-binding proteins.

Biotin-labeled, double-stranded DNA oligonucleotide (oXHW181 plus oXHW198) (50 µg) containing the wild-type (wt) or mutated (c-4) repressor core sequence (oXHW191 plus oXHW199) of HPV18 late promoter was mixed with magnetic streptavidin beads (Pierce; catalog no. 88816) (0.5 ml) that were equilibrated with BC100 buffer (20 mM Tris-Cl [pH 7.9], 100 mM NaCl, 0.5 mM EDTA, 10 mM 2-ME), rotated at room temperature for 45 min, and then washed with BC200 buffer (200 mM NaCl) followed by BC1000 buffer (1,000 mM NaCl). The beads were then resuspended in 0.5 ml of BC200 buffer and incubated at 4°C overnight with 1 ml of HeLa cell nuclear extract preincubated with 0.25 ml of poly(dI-dC)-containing 5× binding buffer (Promega E3050) at room temperature for 30 min. Beads were then washed extensively 6 times with BC200 buffer. The bound proteins were eluted from the beads with BC500 buffer (500 mM NaCl) followed by BC1000 buffer, separated by NuPAGE gel electrophoresis, and visualized by Coomassie blue staining. The protein bands specific to the wt repressor core were excised and then submitted to nano-liquid chromatography-tandem mass spectrometry (LC-MS/MS) performed by a service provider, ProtTech (Phoenixville, PA).

The bound proteins eluted from the beads with BC500 or BC1000 buffer were also denatured in 2× SDS in protein sample buffer containing 5% 2-mercaptoethanol by heating at 90°C for 5 min and were separated in a NuPAGE 4% to 12% bis-Tris gel in 1× NuPAGE MES (morpholineethanesulfonic acid) SDS running buffer (Invitrogen). After being transferred onto a nitrocellulose membrane, the membrane was blocked with 5% nonfat milk–Tris-buffered saline (TBS) for 1 h at room temperature, rinsed with TBS, and incubated overnight at 4°C with a primary antibody. Subsequently, the membrane was washed 3 times with TTBS (TBS with the addition of Tween 20) at a final concentration of 0.1% (vol/vol) and incubated with a horseradish peroxidase-labeled secondary antibody (Sigma) diluted 5,000-fold in TTBS for 1 h at room temperature. After 3 thorough washes with TTBS, the immunoreactive proteins on the membrane were detected with enhanced chemiluminescence substrate (Pierce, Rockford, IL). The signal was captured on X-ray film. Before reprobing with another primary antibody was performed, the membrane was stripped with Restore Western blot stripping buffer (Pierce) according to the manufacturer’s instructions and blocked with 5% nonfat milk–TBS. The primary antibodies used were goat polyclonal anti-hnRNP D0 (T-10) (Santa Cruz) (1:100); mouse monoclonal anti-hnRNP A/B (G-10) (Santa Cruz) (1:100); mouse polyclonal anti-DNA polymerase beta (Abcam, Inc.; catalog no. 2856) (1:500); and anti-β-tubulin (tub 2.1) (Sigma) (1:3,000).

### siRNA and RNA interference (RNAi).

hnRNP A/B siRNA was purchased as an siGENOME SMARTpool from Dharmacon. hnRNP D siRNA and a negative-control siRNA were purchased from Qiagen. HFK18 keratinocytes were transfected twice (before and after a 24-h interval) with a 40 nM concentration of the corresponding siRNA using HiPerFect transfection reagent (Qiagen) according to the manufacturer’s instructions. After 24 h of the second transfection, the cells were lysed with 2× SDS protein gel loading solution and analyzed by Western blotting for the knockdown efficiency of individual proteins as described above.

### Modifying the effect of HPV18 DNA replication by targeting the origin region with a single-strand oligonucleotide.

In *E. coli*, oligonucleotide-mediated recombination depends upon bacterial DNA polymerase participation (53, 54), and the subsequent oligonucleotide-mediated recombination shows a strand bias in which the frequency of recombination is enhanced for one complementary strand over the other (55). We applied a similar oligonucleotide approach to test DNA strand effects on HPV18 DNA replication-mediated late promoter activity. DNA oligonucleotides oXHW391, oXHW392, oXHW393, and oXHW394 (70 nt in length) were cotransfected with plasmid DNA of pXHW15 or pXHW28 into HFK18 cells by electroporation using Amaxa 4-D-Nucleofector according to the protocol described by the manufacturer (Lonza). Approximately 1 × 10^5^ HFK18 cells per nucleofection sample were centrifuged at 200 × *g* for 10 min at room temperature, and the cell pellets were resuspended in 4-D-Nucleofector solution for B lymphocytes at room temperature. Each oligonucleotide (at 10 pM or 5,000 pM) was first mixed with 1 µg pXHW15 or pXHW28 and 75 ng pRL-TS in an Eppendorf tube followed by addition of 20 µl of a mixture of cells with Nucleofector solution, and the resulting mixture was then transferred to the bottom of Nucleocuvette vessels for the nucleofection process. The Nucleocuvette was incubated for 10 min at room temperature after nucleofection, and the transfected cells were resuspended with prewarmed culture medium and plated into each well of 12-well tissue culture plates. After 48 h of incubation at 37°C, cells were harvested and examined for dual luciferase activities using a dual-luciferase reporter assay system (Promega). Relative luciferase activity was calculated by dividing the value representing the light unit readings obtained from a *firefly* luciferase reporter construct by the value representing the light unit readings obtained from the *Renilla* luciferase reporter.

### Small RNA-sequencing (RNA-Seq) analysis.

Small-RNA cDNA libraries from HPV18-infected and uninfected raft tissue samples were prepared as described previously (90). Briefly, 50 µg of total RNA was fractionated on a 15% urea polyacrylamide gel and RNA between the lengths of ~19 nt and ~24 nt was recovered and ligated to a 3′ adapter as follows: first, the ligation products were purified on a 15% denaturing polyacylamide gel; then, small RNAs were recovered and ligated to a 5′ adapter. Ligated small RNAs were purified on a 12% polyacrylamide gel, reverse transcribed using SuperScript III reverse transcriptase (Invitrogen, Carlsbad, CA), and amplified by two rounds of PCR using appropriate primers. All libraries were then sequenced using 454 sequencing, and the sequence reads were aligned against the HPV18 genome.
