# Supplementary figures and images for: Viral DNA Replication Orientation and hnRNPs Regulate Transcription of the Human Papillomavirus 18 Late Promoter
Source: mBio. 2017 May 30;8(3):e00713-17. doi: 10.1128/mBio.00713-17 (PMC5449659; doi:10.1128/mBio.00713-17)

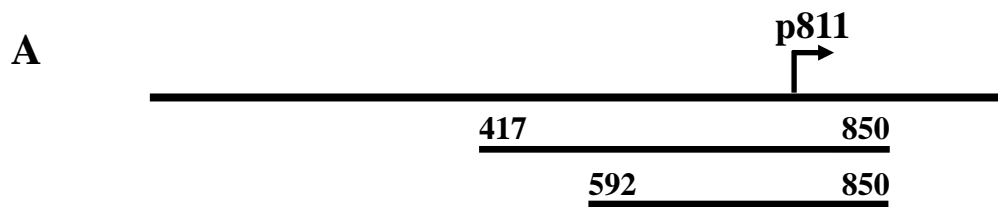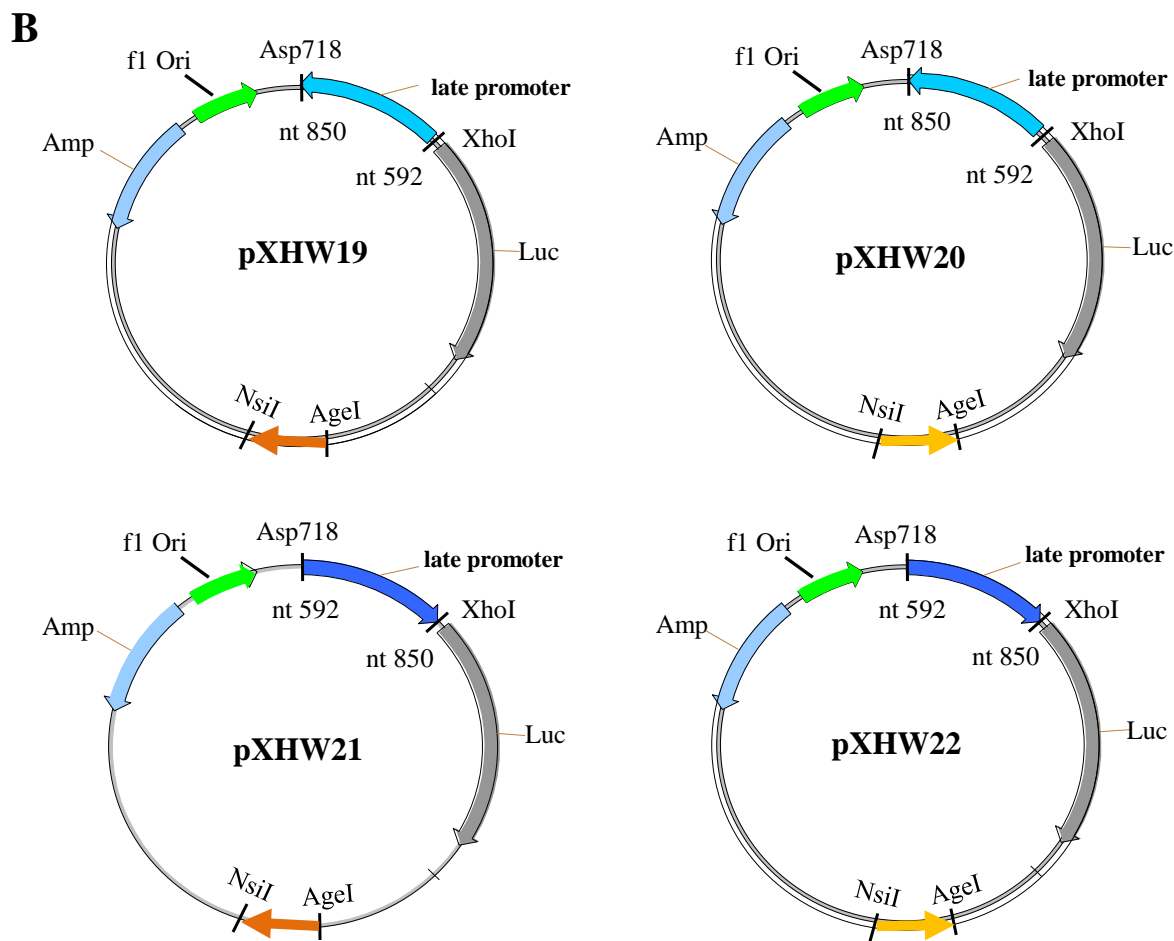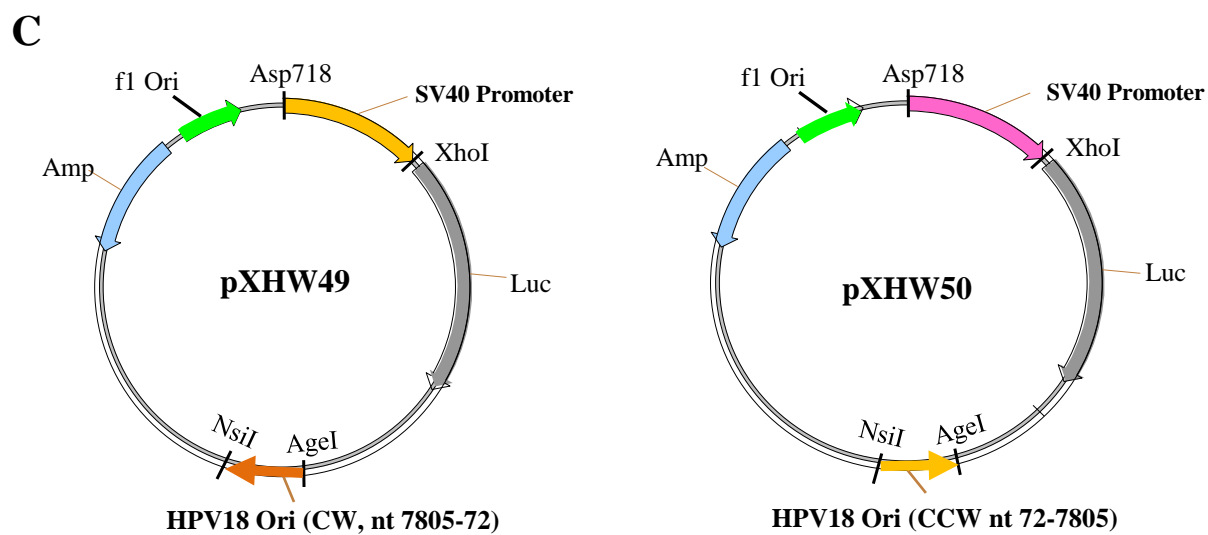

**Fig S1**

**D**

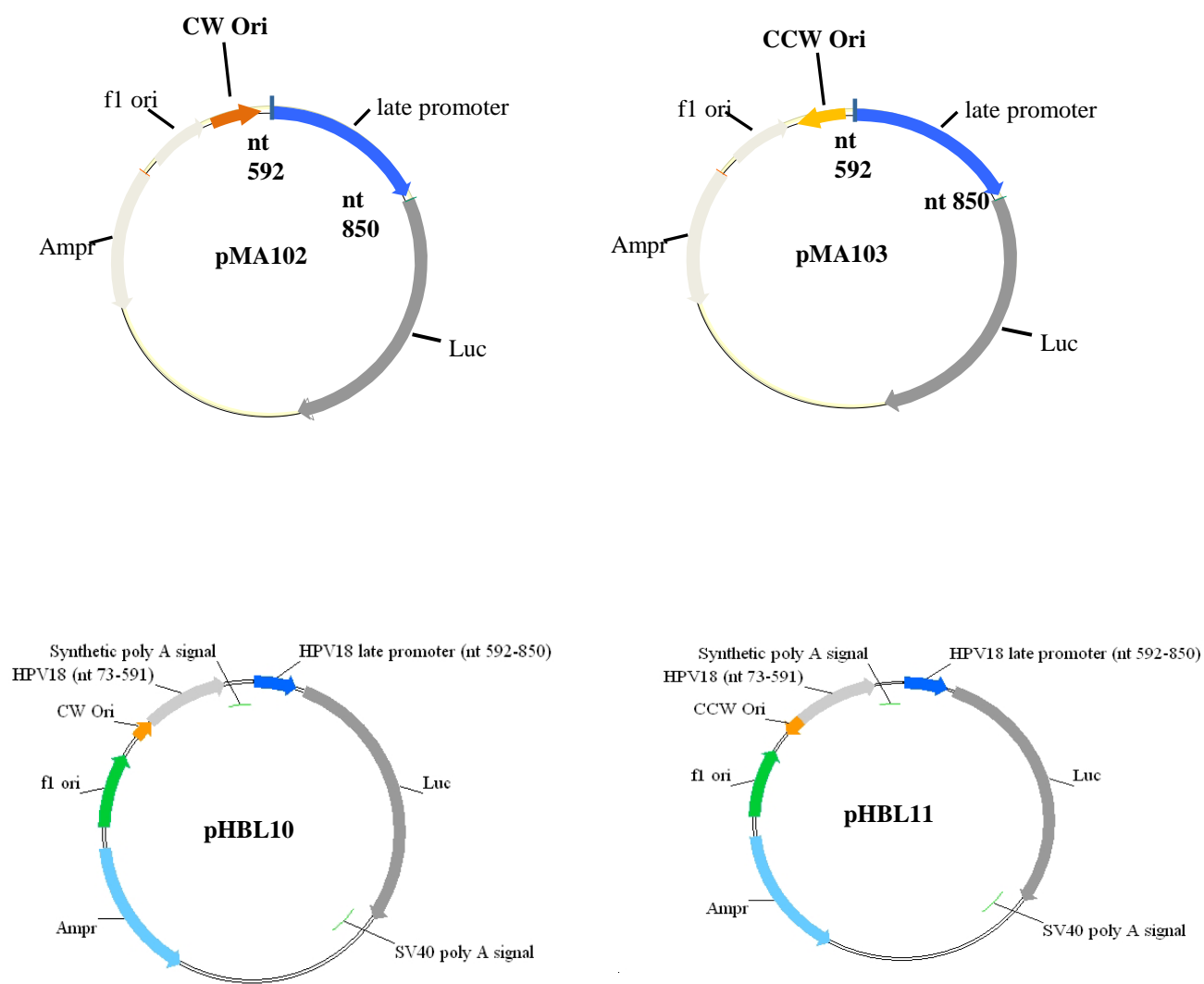

**Fig S1**

Supplement: FIG S1 [file mbo003173324sf1.pdf]

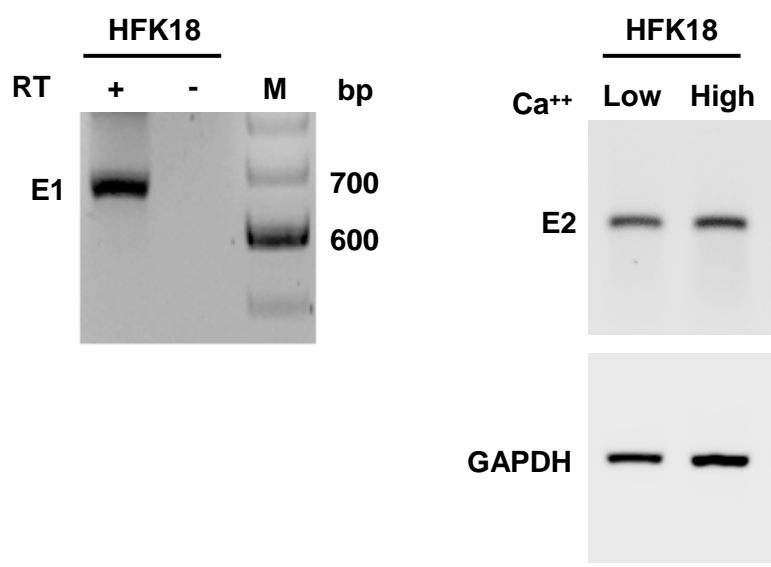

**Fig S3**

Supplement: FIG S3 [file mbo003173324sf3.pdf]

**A**

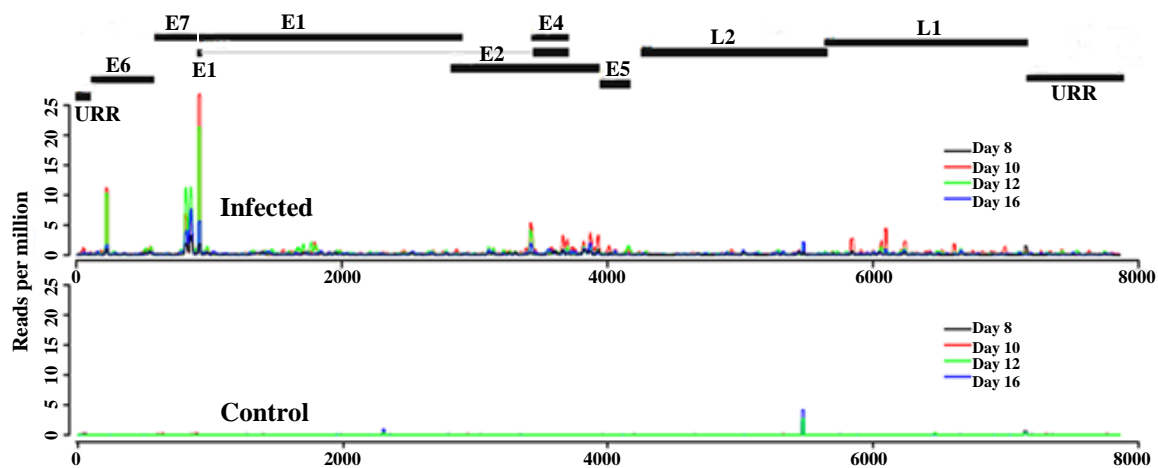

**B**

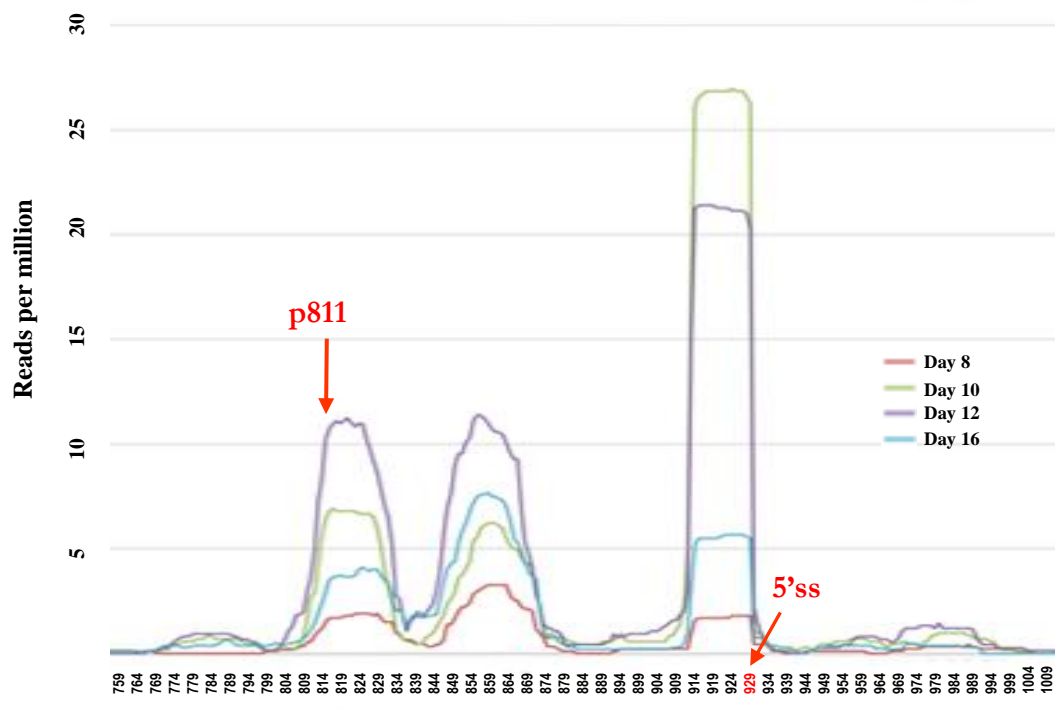

**Fig S4**

Supplement: FIG S4 [file mbo003173324sf4.pdf]
