# Supplementary material for: Viral DNA Replication Orientation and hnRNPs Regulate Transcription of the Human Papillomavirus 18 Late Promoter
Source: mBio. 2017 May 30;8(3):e00713-17. doi: 10.1128/mBio.00713-17 (PMC5449659; doi:10.1128/mBio.00713-17)
Supplement: FIG S2 [file mbo003173324sf2.pdf]

**A**

7805 E2BS3 7857/1

......CTGTGCATACATAGTTTATGCAACCGAAATAGGTTGGGCAGCACATACTATACTTTTC/ATTAATACTTTTAACAATTGTAG

E2BS2

**TATA**TAAAAAAGGGAGTAACCGAAAACGGTC**G**GGACCGAAAACGGTGTA**TATA**AAAGATGTGAGAAACACACCACAAT**A**CT

$P_{55}$ E2BS1 $P_{102}$

105

***ATG****GCGCGCTTTGAGGATCC.......*

**B**

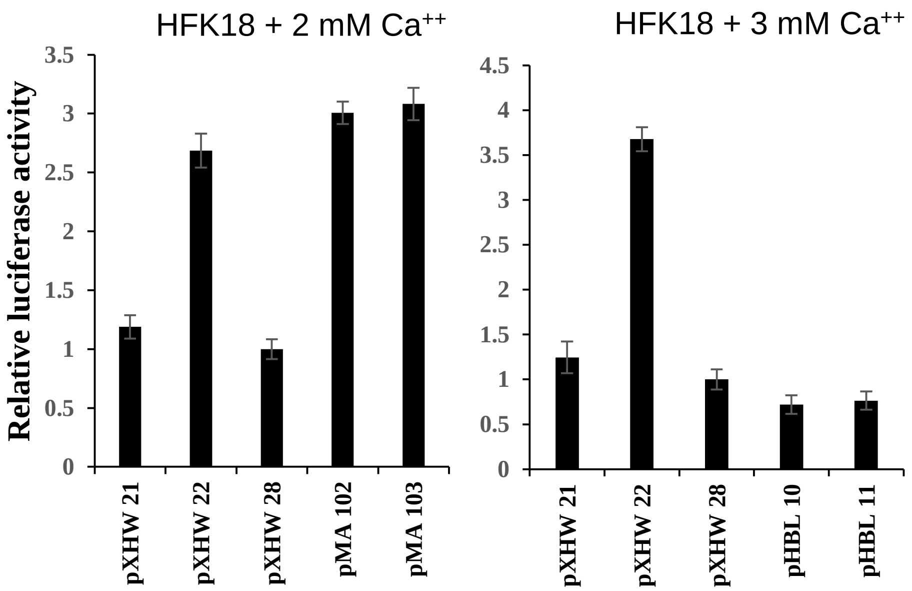


**C**

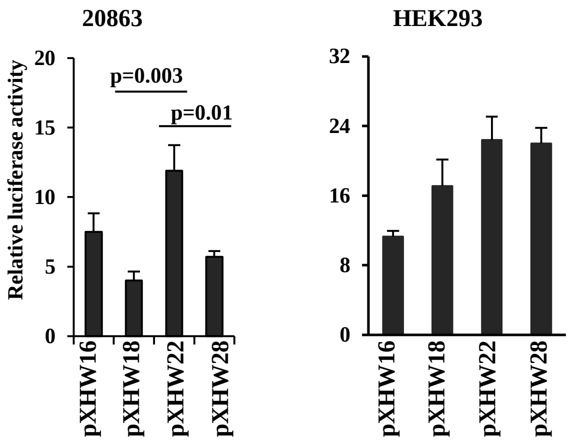


**Fig S2**
