# Supplementary material for: Viral DNA Replication Orientation and hnRNPs Regulate Transcription of the Human Papillomavirus 18 Late Promoter
Source: mBio. 2017 May 30;8(3):e00713-17. doi: 10.1128/mBio.00713-17 (PMC5449659; doi:10.1128/mBio.00713-17)
Supplement: TABLE S1 [file mbo003173324st1.pdf]

Table S1. Peptides identified by LC-MS/MS analysis

Band # 1					
Hits	Protein Mass	No. of Peptide	Sequence Header	gi number	Relative Abundance
1	32119.84	30	>gi|2773158|gb|AAB96683.1| **heterogeneous nuclear ribonucleoprotein D0B [Homo sapiens**]	2773158	79.8%
2	28206.8	10	>gi|31455403|emb|CAD92458.1| aprataxin [Homo sapiens]	31455403	13.0%
3	36059.25	6	>gi|55956919|ref|NP_112556.2| **heterogeneous nuclear ribonucleoprotein A/B isoform a** [Homo sapiens]gi|21757498|dbj|BAC05134.1| unnamed protein product [Homo sapiens]gi|33874222|gb|AAH36708.1| Heterogeneous nuclear ribonucleoprotein A/B [Homo sapiens	55956919	6.4%
4	39010.31	2	>gi|4689144|gb|AAD27781.1|AF077048_1 single strand DNA-binding protein [Homo sapiens]	4689144	0.6%
5	38547.67	2	>gi|7689385|gb|AAF67759.1|AF255675_1 homeoprotein C10 [Homo sapiens]	7689385	0.1%
**Band # 2**					
Hits	Protein Mass	No. of Peptide	Sequence Header	gi number	Relative Abundance
1	31689.75	38	>gi|433344|gb|AAC50056.1| p37 AUF1 [Homo sapiens]	433344	70.5%
2	38432.87	26	>gi|190156|gb|AAA60133.1| **beta-polymerase [Homo sapiens**]	190156	10.7%
3	36059.25	12	>gi|55956919|ref|NP_112556.2| **heterogeneous nuclear ribonucleoprotein A/B isoform a** [Homo sapiens]gi|21757498|dbj|BAC05134.1| unnamed protein product [Homo sapiens]gi|33874222|gb|AAH36708.1| Heterogeneous nuclear ribonucleoprotein A/B [Homo sapiens	55956919	8.8%
4	39616.3	8	>gi|197692437|dbj|BAG70182.1| replication factor C 2 isoform 1 [Homo sapiens]	197692437	1.8%
5	37795.32	8	>gi|119596217|gb|EAW75811.1| deoxynucleotidyltransferase, terminal, interacting protein 1, isoform CRA_b [Homo sapiens]	119596217	2.8%
6	32430.95	8	>gi|14249959|gb|AAH08364.1| Heterogeneous nuclear ribonucleoprotein C (C1/C2) [Homo sapiens]	14249959	3.3%
7	41258.14	4	>gi|62896703|dbj|BAD96292.1| replication factor C 3 isoform 1 variant [Homo sapiens]	62896703	0.5%
8	32687.66	4	>gi|49660012|gb|AAT68294.1| sarcomeric tropomyosin kappa [Homo sapiens]	49660012	0.3%
9	33996.15	2	>gi|119598577|gb|EAW78171.1| replication factor C (activator 1) 4, 37kDa, isoform CRA_b [Homo sapiens]gi|194386378|dbj|BAG59753.1| unnamed protein product [Homo sapiens]	119598577	1.3%
