# Supplementary material for: Viral DNA Replication Orientation and hnRNPs Regulate Transcription of the Human Papillomavirus 18 Late Promoter
Source: mBio. 2017 May 30;8(3):e00713-17. doi: 10.1128/mBio.00713-17 (PMC5449659; doi:10.1128/mBio.00713-17)
Supplement: TABLE S2 [file mbo003173324st2.docx]

| **Primer name** | **HPV18 genome position** | **Orientation** | **Sequence** |
| --- | --- | --- | --- |
| ***Primers used for plasmid construction*** | | | |
| oXHW123 | nt 7805-7825 | Forward | 5’-TAGTTGATCCACCGGT/CATACATAGTTTATGCAACCG-3’ |
| oXHW124 | nt 72-55 | Backward | 5’-GCTGACACAGATGCA/TACACCGTTTTCGGTCCC-3’ |
| oXHW125 | nt 7805-7825 | Forward | 5’-GCTGACACAGATGCAT/CATACATAGTTTATGCAACCG-3’ |
| oXHW126 | nt 71-55 | Backward | 5’-TAGTTGATCCACCGG/TACACCGTTTTCGGTCCC-3’ |
| oXHW127 | nt 417-435 | Forward | 5’-TACTGAGGTACC/TGCCTGCGGTGCCAGAAAC-3’ |
| oXHW128 | nt 850-832 | Backward | 5’-TATTGACTCGAG/CTGGAATGCTCGAAGGTCG-3’ |
| oXHW129 | nt 417-435 | Forward | 5’-TATTGACTCGAG/TGCCTGCGGTGCCAGAAAC-3’ |
| oXHW130 | nt 850-832 | Backward | 5’-TACTGAGGTACC/CTGGAATGCTCGAAGGTCG-3’ |
| oXHW131 | nt 592-609 | Forward | 5’-TACTGAGGTACC/GCATGGACCTAAGGCAAC-3’ |
| oXHW132 | nt 592-609 | Forward | 5’-TATTGACTCGAG/GCATGGACCTAAGGCAAC-3’ |
| oXHW170 | nt 476-495 | Forward | 5’-TACTGAGGTACC/ACGACGATTTCACAACATAG-3’ |
| oXHW171 | nt 530-549 | Forward | 5’-TACTGAGGTAC/CAACCGAGCACGACAGGAAC-3’ |
| oXHW172 | nt 654-673 | Forward | 5’-TACTGAGGTAC/CGGTTGACCTTCTATGTCAC-3’ |
| oXHW173 | nt 711-732 | Forward | 5’-TACTGAGGTACC/TAGATGGAGTTAATCATCAACA-3’ |
| oXHW174 | nt 769-798 | Forward | 5’-GTACC/AATGTTGTGTATGTGTTGTAAGTGTGAAGC/C-3’ |
| oXHW175 | nt 798-769 | Backward | 3’-TCGAG/GCTTCACACTTACAACACATACACAACATT/G-5’ |
| oXHW191 | nt 598-573 | Backward | 5’- TCCATGCAACCTTAATATTATACTTG-3’ |
| oXHW192 | nt 573-598 | Forward | 5’- CAAGTATAATATTAAGTATGTTTGGA-3’ |
| oXHW234 | nt 7805-7825 | Forward | 5’-TACTGAGGTAC/CATACATAGTTTATGCAACCG-3’ goes with oMA411 for pMA102 and pMA103 |
| oHBL39 | nt 7805-7825 | Forward | 5’-CTGAGCGGCCGCC/ATACATAGTTTATGCAACCG-3’ goes with oHBL43 for pHBL10 |
| oHBL40 | nt 7819-7805/73-97 | Forward | 5’-CATAAACTATGTATG/TATAAAAGATGTGAGAAACA-3’ goes with oHBL43 for pHBL11 |
| oHBL41 | nt 87-73/7805-7825 | Forward | 5’-CTCACATCTTTTATA/CATACATAGTTTATGCAACCG-3’goes with oHBL42 for pHBL11 |
| oHBL42 | nt 72-53 | Backward | 5’-CTGAGCGGCCGC/TACACCGTTTTCGGTCCCGA-3’ goes with oHBL41 for HBL11 |
| oHBL43 | nt 591-572 | Backward | 5’-CTGAGCGGCCGC/ATACTTAATATTATACTTGT-3’ goes with oHBL39 for pHBL10 and oHBL40 for pHBL11 |
| oMA411 | nt 72-53 | Backward | 5’-TACTGAGGTACC/TACACCGTTTTCGGTCCC-3’ goes with oMA234 for pMA102 and pMA103 |
|  | SV40 early promoter |  |  |
| oXHW236 | nt 48-69 (pGL3-vector) | Forward | 5’-TACTGAGGTACC/TGCATCTCAATTAGTCAGCAAC-3’ |
| oXHW237 | nt275-258(pGL3-vector) | Backward | 5’-TATTGACTCGA/GCTTTACCAACAGTACCG-3’ |
| *under line indicates enzyme cutting sites: AgeI (ACCGGT), Asp718 (GGTACC), NsiI (ATGCAT), NotI (GCGGCCGC); XhoI (CTCGAG); / lines separate the cutting site from viral sequence. | | | |
| ***Primers used for EMSA assays*** | | | |
| oXHW176 | nt 531-555 | Forward | 5’- AACCGAGCACGACAGGAACGACTCC-3’, probe a |
| oXHW177 | nt 555-531 | Backward | 5’- GGAGTCGTTCCTGTCGTGCTCGGTT-3’, probe a |
| oXHW178 | nt 555-579 | Forward | 5’- CAACGACGCAGAGAAACACAAGTAT-3’, probe b |
| oXHW179 | nt 579-555 | Backward | 5’- ATACTTGTGTTTCTCTGCGTCGTTG-3’, probe b |
| oXHW180 | nt 573-598 | Forward | 5’- CAAGTATAATATTAAGTATGCATGGA-3’, probe c |
| oXHW181 | nt 598-573 | Backward | 5’- TCCATGCATACTTAATATTATACTTG-3’, probe c |
| oXHW182 | nt 800-826 | Forward | 5’- AGAATTGAGCTAGTAGTAGAAAGCTCA-3’, probe d |
| oXHW183 | nt 826-800 | Backward | 5’- TGAGCTTTCTACTACTAGCTCAATTCT-3’, probe d |
| oXHW184 | nt 573-598 | Forward | 5’- CAAGTATGTTATTAAGTATGCATGGA-3’, probe c-1 |
| oXHW185 | nt 598-573 | Backward | 5’- TCCATGCATACTTAATAACATACTTG-3’, probe c-1 |
| oXHW186 | nt 573-598 | Forward | 5’- CAAGTATAATGTTAAGTATGCATGGA-3’, probe c-2 |
| oXHW187 | nt 598-573 | Backward | 5’- TCCATGCATACTTAACATTATACTTG-3’, probe c-2 |
| oXHW188 | nt 573-598 | Forward | 5’- CAAGTATAATATTGTTTATGCATGGA-3’, probe c-3 |
| oXHW189 | nt 598-573 | Backward | 5’- TCCATGCATAAACAATATTATACTTG-3’, probe c-3 |
| oXHW190 | nt 573-598 | Forward | 5’- CAAGTATAATATTAAGGTTGCATGGA-3’, probe c-4 |
| oXHW191 | nt 598-573 | Backward | 5’- TCCATGCAACCTTAATATTATACTTG-3’, probe c-4 |
| oXHW192 | nt 573-598 | Forward | 5’- CAAGTATAATATTAAGTATGTTTGGA-3’, probe c-5 |
| oXHW193 | nt 598-573 | Backward | 5’- TCCAAACATACTTAATATTATACTTG-3’, probe c-5 |
| oXHW194 | nt 573-598 | Forward | 5’- CAAGTATAATATTAAGTATGCAGTTA-3’, probe c-6 |
| oXHW195 | nt 598-573 | Backward | 5’- TAACTGCATACTTAATATTATACTTG-3’, probe c-6 |
| oXHW196 | Human TFIID | Forward | 5’- GCAGAGCATATAAGGTGAGGTAGGA-3’, probe TF |
| oXHW197 | Human TFIID | Backward | 5’- TCCTACCTCACCTTATATGCTCTGC-3’, probe TF |
| oST110 | TBP | Forward | 5’- GTACC/GGCAGCAATATTAAAGGGACC/C-3’, probe TBP |
| oST111 | TBP | Backward | 5’- TCGA/GGGTCCCTTTAATATTGCTGCC/G-3’, probe TBP |
| Promega | Sp1 | Forward | 5’-ATTCGATCGGGGCGGGGCGAGC-3, probe Sp1 |
| Promega | Sp1 | Backward | 5’-GCTCGCCCCGCCCCGATCGAAT-3’, probe Sp1 |
| ***Primers used for southwestern blotting, protein pulldown assays and RT-PCR*** | | | |
| oXHW180 | nt 573-598 | Forward | 5’- CAAGTATAATATTAAGTATGCATGGA-3’, probe c |
| oXHW181 | nt 598-573 | Backward | 5’- TCCATGCATACTTAATATTATACTTG-3’, probe c |
| oXHW191 | nt 598-573 | Backward | 5’- TCCATGCAACCTTAATATTATACTTG-3’, probe c-4 |
| oXHW198 | nt 573-598 | Forward | 5’- Biotin-CAAGTATAATATTAAGTATGCATGGA-3’, probe c |
| oXHW199 | nt 573-598 | Forward | 5’- Biotin-CAAGTATAATATTAAGGTTGCATGGA-3’, probe c-4 |
| oZMZ252 | nt 121-140 | Forward | 5’-ATCCAACACGGCGACCCTAC-3’ for E1 detection by RT-PCR |
| oZMZ 229 | nt 967-948 | Backward | 5’-CTGAGTCGAC/AAACCAGCCGTTACAACCCG-3’ for E1 detection by RT-PCR |
| oXHW46 | nt 822-840 | Forward | 5’-GCTCAGCAGACGACCTTCG-3’ for E2 detection by RT-PCR |
| oMA97 | nt 2867-2848 | Backward | 5’-GTCCTGCAACGCACTTAAAC-3’for E2 detection by RT-PCR |
|  | | | |
| ***Oligos used for targeting the plasmid regions upstream or downstream of HPV18 Ori in HFK18 cells*** | | | |
| oXHW391 | nt 2679-2748 in pGL3-basic vector | Forward | 5’-GGTAACAGGATTAGCAGAGCGAGGTATGTAGGCGGTGCTACAGAGTTCTTGAAGTGGTGGCCT  AACTACG-3’ |
| oXHW392 | nt 2748-2679 in pGL3-basic vector | Backward | 5’-CGTAGTTAGGCCACCACTTCAAGAACTCTGTAGCACCGCCTACATACCTCGCTCTGCTAATCCTG  TTACC-3’ |
| oXHW393 | nt 2422-2491 in pGL3-basic vector | Forward | 5’-CTGGAAGCTCCCTCGTGCGCTCTCCTGTTCCGACCCTGCCGCTTACCGGATACCTGTCCGCCTTTC  TCCC-3’ |
| oXHW394 | nt 2491-2422 in pGL3-basic vector | Backward | 5’-GGGAGAAAGGCGGACAGGTATCCGGTAAGCGGCAGGGTCGGAACAGGAGAGCGCACGAGGGA  GCTTCCAG-3’ |
